# Expressing intrinsically-disordered tardigrade proteins has positive effects on acute but not chronic stress tolerance in *Saccharomyces cerevisiae*

**DOI:** 10.1371/journal.pone.0325682

**Published:** 2025-06-06

**Authors:** Mario León López, Ian Wheeldon, Joshua T. Morgan

**Affiliations:** 1 Department of Bioengineering, University of California, Riverside, California, United States of America; 2 Department of Chemical and Environmental Engineering, University of California, Riverside, Riverside, California, United States of America; 3 Center for Industrial Biotechnology, University of California, Riverside, California, United States of America; Federal University Dutse, NIGERIA

## Abstract

The production of high value and commodity chemicals, biopharmaceuticals and biofuels using *Saccharomyces cerevisiae* is hindered by various stress factors that affect yield and efficiency. Tardigrades, known for their remarkable stress tolerance, express unique proteins responsible for their resilience. This study evaluates the impact of expressing the tardigrade proteins CAHS3, MAHS, and RvLEAM on stress tolerance in *S. cerevisiae*. Our results show that high yields of these proteins do not impede yeast growth, except for CAHS3, which reduces proliferation. Expression of MAHS enhances acute heat tolerance, while MAHS and RvLEAM confer increased tolerance to acute hyperosmotic stress. Both CAHS3 and RvLEAM improve desiccation survival. However, these proteins do not provide benefits under chronic stress conditions such as prolonged exposure to high temperature, hyperosmotic stress, or solvents. These findings highlight the potential utility of tardigrade proteins for transient stress protection in industrial bioprocesses and suggest future engineering approaches for improved stress tolerance in yeast.

## Introduction

*Saccharomyces cerevisiae* is widely recognized for its industrial applications, particularly in the production of biofuels, biochemicals, heterologous proteins, and substances of biopharmaceutical interest [1–3]. Studies highlight its use as a heterologous host for natural products, capitalizing on its robustness, ease of genetic engineering, and vast research background, features that contribute to its success as a cell factory [4–6]. As one prominent example, the yeast’s efficiency in bioethanol synthesis addresses the global demand for renewable and sustainable fuels, presenting an attractive alternative to conventional fossil fuels [7–9]. The biosynthesis process aligns with broader initiatives for renewable fuels and chemicals, crucial for addressing environmental concerns and reducing dependence on non-renewable petroleum-based fuels [7,10,11].

In the context of industrial processes such as biochemical production or heterologous protein expression, stress tolerance becomes a critical aspect. For example, biofuel and biochemical production involve stressors, such as high heat, hyperosmotic stress and formation of alcohol byproducts (e.g., glycerol or acetic acid), to which wildtype *S. cerevisiae* is not resistant at high concentrations. In terms of heat tolerance, the optimal temperature for the growth of *S. cerevisiae* is between 28°C and 35°C, while some of the enzymes used for the breakdown of (ligno-)cellulose have an optimal temperature between 45°C and 55°C; a thermotolerant yeast species that thrive at temperatures of 40°C or higher are desired to increase efficiency and reduce the cooling expenses and the associated risk of contamination [12,13]. Biofuel and biochemical production also involves hyperosmotic pressure, due to high concentrations of sodium salts used during the pretreatment of the (ligno-)cellulosic material or due to elevated sugar concentrations required for high gravity fermentation [13]. Alcohol products of biofuel production, such as propanol, n-butanol, isopentanol and ethanol, are toxic for yeast through various mechanisms; one key mechanism is solvent mediated membrane disruption [14]. These stressors hinder the bioethanol and biofuel production capabilities of *S. cerevisiae* thus prompting systems biology and metabolic engineering research to address these challenges [13–15]. These approaches integrate traditional methods with synthetic biology, aiming to enhance stress tolerance in *S. cerevisiae* strains [16,17]. Notably, the engineering of *S. cerevisiae* for efficient fermentation of cellulose in biofuel production is a significant research area [8]. Processes involving biomass pretreatment, enzymatic hydrolysis of polysaccharides, and fermentation of resulting sugars, especially in consolidated bioprocess, showcase the yeast’s robustness and adaptability [18]. Challenges remain in heterologous expression of cellulolytic enzymes and co-fermentation of different sugar types, underscoring the importance of stress tolerance in optimizing these processes for industrial viability [10,19,20].

Tardigrades (“water bears”) are microscopic animals that possess extraordinary stress tolerance, including extended survival during complete desiccation and freezing [21,22]. While tardigrade physiology is not completely understood, recent research has identified proteins contributing to their resistance. A number of tardigrade-unique proteins, including tardigrade-specific desiccation-associated proteins (TDAPs) and Late embryogenesis abundant (LEA) proteins, have been linked to their ability to resist the stress of total desiccation, and are speculated to stabilize protein and membrane structure [23–25]. There are tardigrade variants which accumulate in the cytosol (cytosolic abundant heat soluble; CAHS), localize to the mitochondria (mitochondria abundant heat soluble protein (MAHS) and *Ramazzottius varieornatus* mitochondrial LEA; RvLEAM), as well as other sites [23,26,27]. Several studies have shown TDAP localization is preserved in other species [26–33]. CAHS has been shown to perform a chaperone-like role in maintaining protein structure and function *in vitro* and, in bacteria and/or yeast systems, although these studies focused specifically on desiccation tolerance [23]. Both MAHS and RvLEAM increase human cell survival after hyperosmotic stress [27]. Further, MAHS has been shown to increase human cell solvent tolerance [34], while CAHS expression increases solvent tolerance of cyanobacterium [35].

Here, we evaluate the hypothesis that tardigrade proteins, specifically CAHS3, MAHS, and RvLEAM, protect proliferating yeast against relevant stressors when constitutively expressed. We present data showing constitutive expression of CAHS3, MAHS, and RvLEAM results in expected localization in yeast and does not halt proliferation. Further, we show MAHS protects against acute (but not chronic) heat stress, while both MAHS and RvLEAM protect against acute osmotic stress. Finally, we confirm prior results of CAHS3 improving desiccation tolerance in yeast while extending these results to RvLEAM. In aggregate, these data demonstrate that tardigrade proteins provide context dependent protection in yeast, meriting further investigation.

## Results

### Tardigrade proteins localize as expected in *S. cerevisiae*

**Fig 1:**
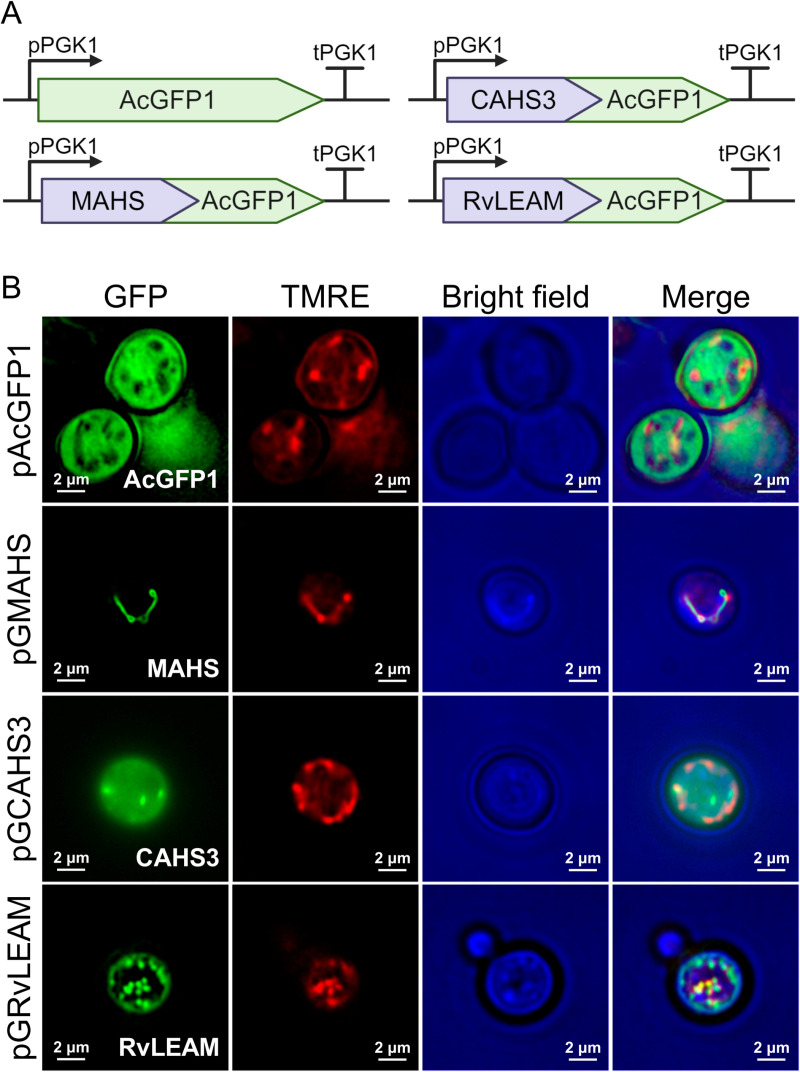
Expression of tardigrade transgenes in *S. cerevisiae.* (A) DNA constructs used in the expression and localization of GFP-fused tardigrade proteins. (B) Representative fluorescence microscopy images of S. cerevisiae expressing AcGFP1 as a cytosolic control, MAHS-AcGFP1, CAHS3-AcGFP1 and RvLEAM-AcGFP1 (green). Mitochondria are identified with TMRE (red), which stains live mitochondria. Images were taken using a 100x objective and merged images of green fluorescence, red fluorescence, and brightfield contrast (blue) are shown. Colocalization of red and green fluorescence shows in yellow.

GFP-tagged tardigrade proteins were cloned into an existing 2μ pRS426 backbone with expression under a constitutive yeast (PGK1) promoter (shown schematically in Fig 1A), similar to prior work [36]. Tardigrade-protein-expressing yeast were imaged to assess cellular localization. In other systems, CAHS3 localizes to the cytosol, while MAHS and RvLEAM localize to the mitochondria. In representative images we show similar localization in *S. cerevisiae* (Fig 1B). MAHS and RvLEAM colocalized with Tetramethylrhodamine ethyl ester perchlorate (TMRE), a mitochondrial stain, while CAHS3 localized throughout the cell. In some cases, we observed CAHS3 localizing in puncta (Fig 1B), with this more frequent with higher expression (Fig S1 in S1 File).

### *S. cerevisiae* remains proliferative after expression of tardigrade proteins

**Fig 2:**
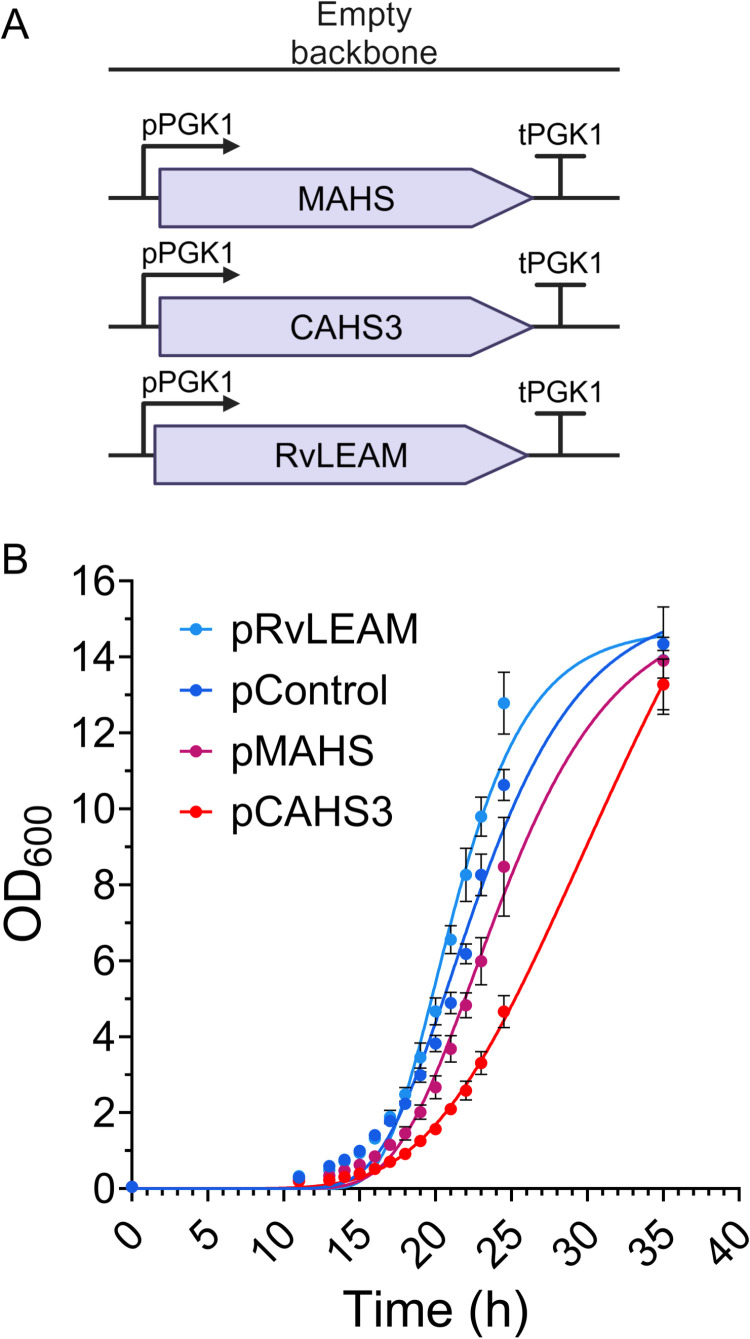
Plasmid constructs used and the effect of the tardigrade proteins on cell growth. (A) Graphic illustration of the transcriptional units assembled in the 2μ plasmid that were used for stress tolerance testing. (B) Growth curves of the yeast strains, three of them expressing tardigrade proteins (pRvLEAM, pMAHS, pCAHS3) and one carrying an empty vector (pControl).

Yeast containing an empty vector (pControl), plasmid expressing MAHS (pMAHS), CAHS3 (pCAHS3) or RvLEAM (pRvLEAM) (Fig 2A) were grown at 30°C to determine the effect of the heterologous proteins in the growth of our yeast strains. Culture OD_600_ was measured at 0 h, 11 h and 13 h, and then every hour to 24 h point (to capture the log phase of growth) with a last measurement at 35 h. Growth curves were fit to a modified Gompertz model as previously described [37,38]; maximum growth *μ* was used to statistically compare growth rates. pControl was used as a control (*μ* = 0.342 ± 0.013) and significance was assessed via one-way ANOVA followed by Dunnett’s post-hoc test. pRvLEAM grew faster (*μ* = 0.383 ± 0.001; *p* = 0.0002) than pControl while both pMAHS (*μ* = 0.321 ± 0.005; *p* = 0.014) and pCAHS3 (*μ* = 0.273 ± 0.003; *p* < 0.0001) grew significantly slower (Fig 2B).

### MAHS expression increases *S. cerevisiae* acute heat tolerance

**Fig 3:**
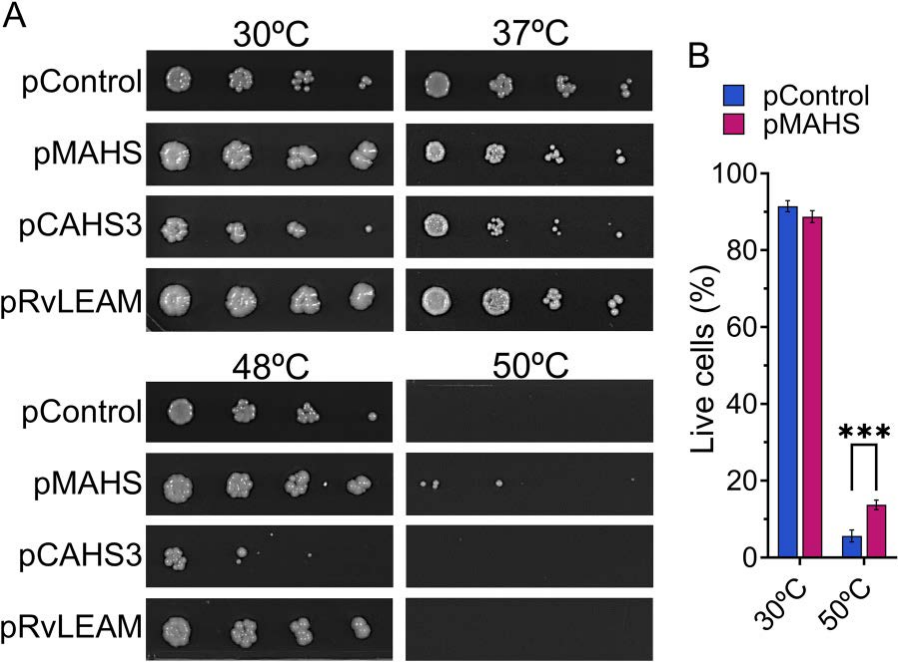
Effect of the tardigrade proteins on cell viability after heat shock. (A) Representative images of a 3-day growth of the different plasmid-carrying yeast strains spot-plated after 1 h heat stress in a 5-fold dilution starting at an OD_600_ of 0.5. Showing pMAHS as the lone survivor at 50°C. (B) Live/Dead cytometry assay for pMAHS and pControl analyzed using two-way ANOVA with a Bonferroni correction (n = 3).

We then proceeded to determine the impact of tardigrade gene expression on stress tolerance in *S. cerevisiae*. Two biological replicates of each protein-expressing yeast were heat shocked at temperatures ranging from 30°C (control condition) to 50°C for 1 h prior to making serial dilutions and plating on 3 plates of selective media (Fig S2 in S1 File; representative images shown in Fig 3A). Consistent with the growth curve results, pCAHS3 expression resulted in reduced population in all test conditions. pMAHS and pRvLEAM performed equivalently to or more robustly than pControl at 30°C, 37°C, and 48°C; however, only pMAHS expressing cultures exhibited improved survival at 50°C. To confirm these findings, the 50°C heat shock was repeated with pControl and pMAHS expressing cultures, followed by a Live/Dead assay (Fig 3B). pMAHS demonstrated significantly higher viability than pControl (*p* = 0.0003) after 50°C heat shock.

### RvLEAM or MAHS expression improves while CAHS3 expression impairs *S. cerevisiae* hyperosmolarity tolerance

**Fig 4:**
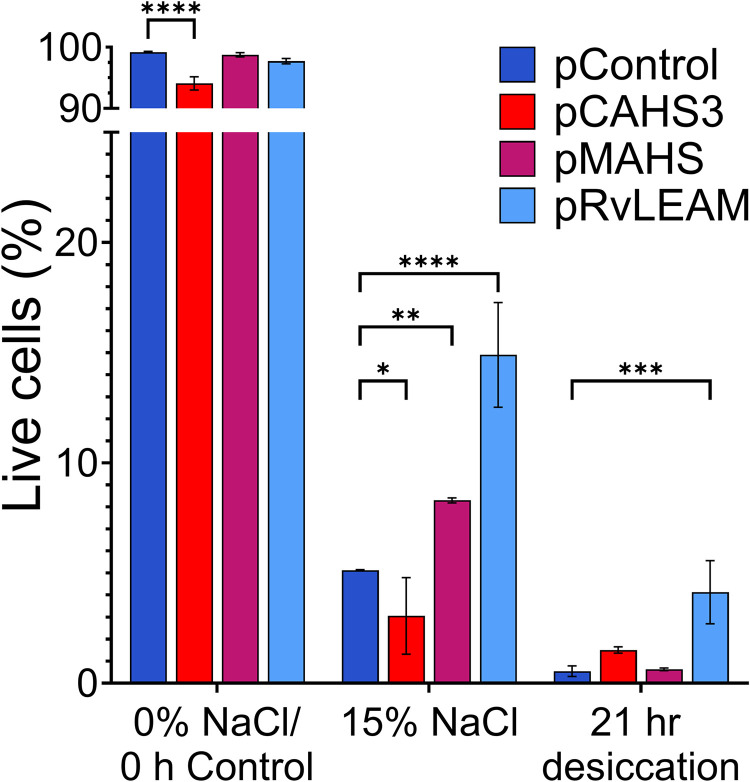
Effect of tardigrade proteins in desiccation and hyperosmolarity acute stress. Yeast expressing CAHS3, MAHS or RvLEAM, as well as BY4741 containing an empty vector (pCAHS3, pMAHS, pRvLEAM and pControl respectively) were subjected to desiccation and hyperosmolarity stress prior to Live/Dead assay. Stress conditions were performed simultaneously and shared the control condition. Live/Dead assay showed significant stress tolerance improvement for pRvLEAM in both stress conditions and improvement for pMAHS under hyperosmolarity stress (n = 3).

In order to test the effect of tardigrade gene expression on tolerance to desiccation and hyperosmolarity, our strains were grown overnight from an OD_600_ of 0.1 (0.2 for pCAHS3) and diluted in water to a consistent OD_600_ of 0.5 in 1 mL prior to centrifuging for 5 min at 3000 × *g*. After centrifuging, the supernatant was discarded, and the pellets were resuspended in 1mL of mSC -Ura media for the control, or in 15% NaCl mSC -Ura media for hyperosmotic stress. For desiccation, the pellets were left to dry in open tubes covered with a 3M micropore tape. All three conditions were incubated then in a shaking incubator at 30°C for 21 h. Expression of CAHS3 significantly reduced viability under basal (0 h/0% NaCl Control) and hyperosmotic (15% NaCl) conditions (Fig 4, p < 0.0001 and p = 0.0498, respectively); but had no significant difference compared to control for desiccation. This is consistent with the growth curve and heat shock data shown previously in Fig 2B and Fig 3A. Expression of MAHS improved 15% NaCl hyperosmotic stress tolerance (p = 0.0024) but did not increase viability after complete desiccation. On the other hand, RvLEAM conferred significant tolerance to both 15% NaCl hyperosmotic stress (*p* < 0.0001) and desiccation (*p* = 0.0007).

### Selected tardigrade proteins do not confer tolerance to chronic stressors in *S. cerevisiae*

**Fig 5:**
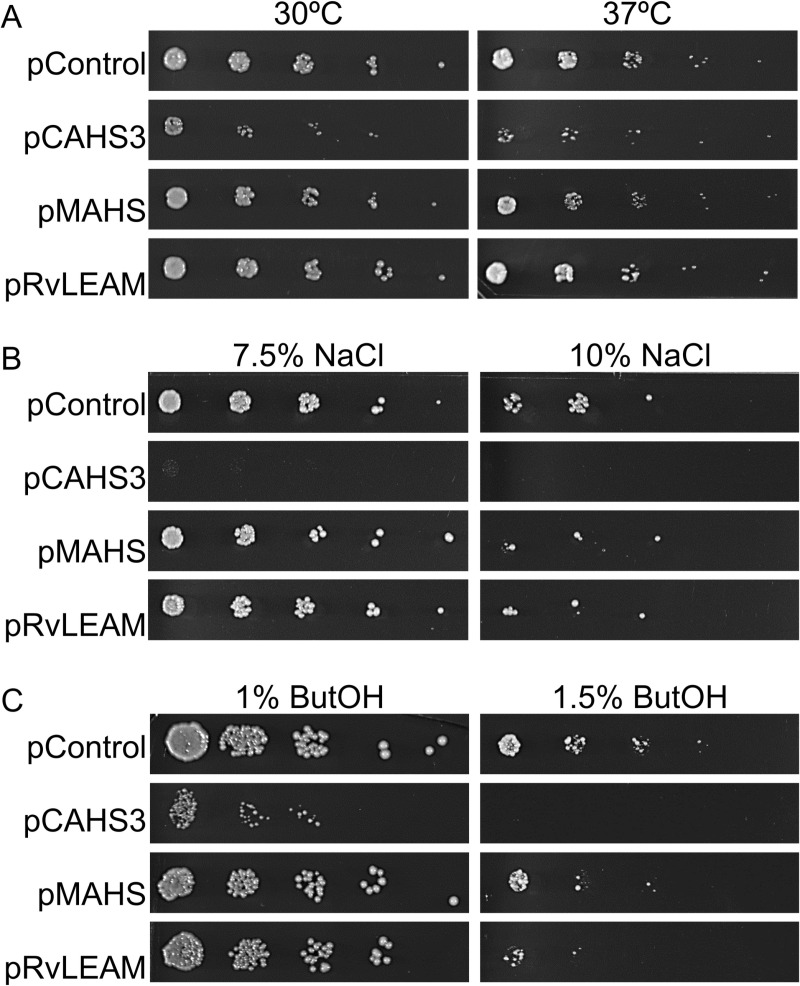
Spot plate growth under chronic stress. (A) Representative images of the different plasmid-carrying yeast strains spot-plated in a 5-fold serial dilution starting at an OD_600_ of 0.5 after 2 days of growth at 30°C and 37°C. (B) Representative images of the different plasmid-carrying yeast strains spot-plated as in (A) after 6 days of growth in 7.5% NaCl, and after 9 days of growth in 10% NaCl. (C) Representative images of the yeast on ButOH after 5 days in 1% ButOH, and after 8 days in 1.5% ButOH exhibiting lack of better growth in the tardigrade protein expressing strains than the control pControl.

While the prior results indicate tardigrade gene expression may provide benefit to acute stress, we wished to determine the benefits under chronic conditions relevant to the bioethanol and biofuel industry. pControl and tardigrade protein expressing strains were subjected to chronic thermal, hyperosmotic, or solvent stressors. In all cases cells were grown overnight to log phase, and then serially diluted 5-fold in a 96-well plate. Dilutions were transferred onto an mSC -Ura plate with a 96-well pin replicator. For heat stress, the plates were incubated at 30°C, 37°C and 40°C for 2 days. Cells grew to some degree on every temperature except 40°C, but none of them grew better than the control, pControl (Fig 5A; 40°C data shown in Fig S3B in S1 File).

For hyperosmotic stress, cells were transferred onto mSC -Ura containing a range of NaCl concentrations of 2.5% increments from 5% to 12.5%. In Fig 5B, 7.5% and 10% NaCl were chosen as representative images showing some effect on growth, pCAHS3 did not grow starting at 7.5% NaCl, and nothing grew at a NaCl percentage higher than 10%, which took 9 days to grow. When testing for solvent tolerance, cells were transferred onto mSC -Ura plates containing n-butanol (ButOH) at concentrations that ranged between 0.5% and 2.5% at 0.5% increments, 1% and 1.5% ButOH were chosen as representatives of the experiment result. As shown in Fig 5B, none of the tardigrade proteins provided an enhanced solvent tolerance in presence of ButOH, and cells would not grow at 2% ButOH or higher. pCAHS3 did not grow well past 0.5% ButOH (Fig S3C in S1 File).

## Discussion

Here, we have demonstrated that expressing tardigrade proteins can offer protection in *S. cerevisiae* to some acute stresses and desiccation, but not to chronic presentation of stress. This adds to prior data on the expression of tardigrade proteins in yeast for desiccation [23], and further demonstrates acute tolerance to stressors associated with bioproduction. First, we show that GFP-tagged versions of CAHS3, MAHS, and RvLEAM localize as expected in *S. cerevisiae*. Second, we demonstrate that expressing MAHS and RvLEAM does not halt *S. cerevisiae* proliferation, although CAHS3 significantly decreases it. Third, we demonstrate that MAHS expression is protective against acute heat shock. Fourth, we demonstrate that MAHS and RvLEAM expression provide protection to acute salt, desiccation and heat shock. Finally, we demonstrate that expressing CAHS3, MAHS, or RvLEAM does not provide any benefits to chronic exposure to heat, salt, or solvent (ButOH) stress. These studies motivate further investigations into extremophile protein mediated stress tolerance in production cell strains.

An important consideration when interpreting these results is the choice of backbone. The tardigrade proteins were expressed with a PGK1pr on a 2μ plasmid, resulting in high overall expression; high expression of tardigrade proteins might have had detrimental behavior due to excess protein. Specifically, we observed that higher levels of CAHS3 protein correlated with the formation of fluorescent structures in the cells (Fig S1 in S1 File). CAHS3 has been shown to polymerize and bind to actin in mammalian cells [28,29], this may contribute to aggregate formation or the observed proliferation defect. Overall, CAHS3 expression exhibited toxicity when compared to the expression control, future studies would benefit from assessing expression level with regard to CAHS3. Beyond possible interactions with the cytoskeleton, CAHS3’s aggregates may also affect core metabolic processes. By altering cytoplasmic organization, CAHS3 could hinder diffusion-limited metabolic reactions, passively sequester key metabolic enzymes, or interfere with organelle and vesicle intracellular trafficking. Additionally, its interaction with actin may also impair mitochondrial positioning, potentially reducing energy distribution efficiency. These combined effects could contribute to the observed proliferation defect. Future studies using metabolomics or flux analysis may help clarify these potential impacts.

The distinction between acute and chronic expression of stress in this study has relevance for bioproduction. To use biofuel production as an example, there is benefit to yeast that can ferment at higher temperatures [13], salt concentrations [13], as well as resist solvents produced during fermentation [14,39,40]. Unfortunately, our current results suggest that tardigrade protein expression in yeast is not a solution to these challenges, although CAHS has shown benefit in other model organisms [35]. However, tolerance to acute heat and salt stress may provide benefits in ethanol production after heat shock, which has been proven to increase glycerol and ethanol yields [41,42]; or during high ethanol productivity in a two-stage bioreactor that requires high salt concentrations [43].

The mechanisms of protection of CAHS3, MAHS, and RvLEAM is incompletely understood; mechanism is not directly addressed in this work. However, tardigrade transgenes have been demonstrated as functional protection in yeast, bacteria, mammalian cells, and *in vitro* [23,24,27,30,44]; however, the results are not uniformly positive [45]. Specifically, MAHS and RvLEAM have previously been demonstrated to confer hyperosmotic tolerance [27], which is related to desiccation tolerance. Further, it has been hypothesized that MAHS may protect mitochondria through increasing oxidative stress tolerance, reducing oxidative stress, or maintaining mitochondrial membrane integrity [25]. Importantly, MAHS possesses an α-helix sequence similar to PsLEAM a plant LEA protein, which is known to stabilize membranes [46]. RvLEAM also possesses a similar sequence, suggesting it may work via membrane stabilization as well [27]. This could be related to the effect RvLEAM has on the size and shape of the mitochondria, making them appear bigger and somewhat spherical (Fig 1B and Fig S1 in S1 File), although this was not quantified. This shape has been suggested to be potentially linked to a loss of membrane potential [47], which could be caused by an increase in the positive charges in the mitochondrial matrix due to the charge of RvLEAM at physiological conditions (pI ~ 9.7). It has been shown previously that mitochondria attracts cationic proteins, such as K-Ras and Ras-like proteins, due to an increase in their negative charge during early apoptosis [48–50]. Therefore, we hypothesize that a more electropositive mitochondria, along with a more stable membrane, might delay apoptosis under acute stress. While not directly tested in these studies, our findings are consistent with these hypothesized mechanisms. This may help explain why mitochondrial-targeted proteins such as MAHS and RvLEAM have a more pronounced protective effect than cytosolic proteins like CAHS3. Mitochondria are essential for cellular energy production and cell survival because mitochondrial damage often triggers apoptosis and leads to irreversible cell death [47,48]. Especially under acute stress, maintaining mitochondrial integrity may be particularly critical for overall cell viability, as proteins that preserve mitochondrial function can confer a disproportionately greater benefit to cell survival than those acting in the cytosol.

As mentioned above, the protective mechanisms of CAHS are incompletely understood. CAHS has been shown to vitrify and may contribute to the stable vitrification of tardigrades during desiccation [51], although it remains unclear if this is the dominant mechanism of anhydrobiosis [51,52]. Further, CAHS has been shown to reversibly form supportive filamentous networks under hyperosmotic stress, increasing cell stiffness and potentially improving survival through mechanical stabilization of the cell [29]. Importantly, CAHS proteins are a relatively broad family that is conserved within tardigrades [26], several CAHS proteins have been demonstrated to individually increase the desiccation tolerance of yeast and bacteria [23]. Of these, we only investigated CAHS3 from *R. varieornatus*, CAHS variants likely have some diversity of function. A recent study of several CAHS variants of another tardigrade species (*H. exemplaris*) demonstrated that the protective effect of several CAHS variants are synergistic with trehalose, and CAHS alone offered limited protection to desiccation stress [53]. Trehalose-6-phosphate synthase is expressed by *R. varieornatus* [54]; however, it is unknown if trehalose has synergistic activity with CAHS3 specifically. If so, it is possible that the addition or synthesis of trehalose would augment the effects of CAHS3 expression in yeast.

There are several limitations of these studies to keep in mind. A key limitation of the study is that the experiments were largely conducted at mid-log phase since cells become highly tolerant to stress during stationary phase [55–57]. In bioproduction, cells are not restricted to log phase, a larger experimental design would assess these proteins at stationary phase as well. While amenable to genetic studies, the strain used (BY4741) is a derivative of S288C, which has a mutated copy of HAP1, a gene that locates to the mitochondria under stress [58]. Further, these studies only expressed a single transgene at a time; in native tardigrade these genes and others are co-expressed and may require other tardigrade proteins to function. Indeed, it has previously been speculated that MAHS and RvLEAM may have complimentary function [27]. Finally, the significant reduction in proliferation of CAHS3 expressing cells (Fig 2) impacts the assessment of our screening. While all experiments have consistent OD_600_ values and were initiated in the log phase, qualitative assessment based on colony size may be impacted by altered proliferation, although not the quantitative Live/Dead studies (Fig 4).

Overall, we have demonstrated stable expression and localization CAHS3, MAHS, and RvLEAM in a representative production cell line, *S. cerevisiae*. Further, we demonstrated modified stress tolerance of *S. cerevisiae* via transgenic extremophile protein expression in specific contexts. Importantly, resistance to acute heat shock was demonstrated with MAHS expression, not previously shown with yeast. As an important limitation to this approach, we showed CAHS3 expression negatively impacts proliferation and viability. Future studies testing expression level, inducible expression, and multiple gene expression are warranted. Further, as expression of heterologous transgenes may impact cell phenotype in unexpected ways, testing in production environments will be critical.

## Materials and methods

### Strains and culture conditions

Strains used are shown in Table 1. *Escherichia coli* (*E. coli*; NEB10β; New England Biolabs, Ipswich, MA) was used for cloning which were grown in Lysogeny Broth (LB) with ampicillin (5 g/L yeast extract, 10 g/L tryptone, 10 g/L NaCl, 100 mg/L ampicillin; Research Products International (RPI), Mount Prospect, IL, USA). *S. cerevisiae* strains without plasmids were grown in YPD media containing 20 g/L peptone, 10 g/L yeast extract (RPI, Mount Prospect, IL, USA) and 20 g/L glucose (Fisher Scientific, Waltham, MA, USA) in a container adequate for each experiment. *S. cerevisiae* strains containing plasmids were grown in Synthetic Complete medium minus uracil (SC -Ura) which contained 1.7 g/L Yeast Nitrogen Base without Amino Acids and Ammonium Sulfate (YNB; Becton, Dickinson and Company, Franklin Lakes, NJ, USA), 5 g/L Ammonium Sulfate (RPI, Mount Prospect, IL, USA), 2 g/L of handmade synthetic dropout mix without uracil (0.25 g Adenine, 1 g Alanine, 1 g Arginine, 1 g Asparagine, 1 g Aspartic acid, 1 g Cysteine, 1 g Glutamine, 1 g Glutamic acid, 1 g Glycine, 1 g Histidine, 1 g Inositol, 1 g Isoleucine, 5 g Leucine, 1 g Lysine, 1 g Methionine, 0.1 g para-Aminobenzoic acid, 1 g Phenylalanine, 1 g Proline, 1 g Serine, 1 g Threonine, 1 g Tryptophan, 1 g Tyrosine, 1 g Valine; RPI, Mount Prospect, IL, USA), and 20 g/L glucose (Fisher Scientific, Waltham, MA, USA). When necessary, solid media were prepared as above with 20–30 g/L agar (Fisher Scientific, Waltham, MA, USA). All transformed yeast cultures were grown at 30 °C, while *E. coli* cultures for molecular cloning were grown at 37°C.

**Table 1:** Strains used for molecular biology and stress testing.

Strains	Genotype	Source
*E. coli*		
NEB10β	*Δ(ara-leu) 7697 araD139 fhuA ΔlacX74 galK16 galE15 e14- ϕ80dlacZΔM15 recA1 relA1 endA1 nupG rpsL (StrR) rph spoT1 Δ(mrr-hsdRMS-mcrBC)*	New England Biolabs
*S. cerevisiae*		
BY4741	*MATa his3Δ1 leu2Δ0 met15Δ0 ura3Δ0*	Open Biosystems (YSC1048)
pAcGFP1	*BY4741 + pRS426-P*_*PGK1*_ *-AcGFP1-T*_*PGK1*_	This study
pCAHS3	*BY4741 + pRS426-P*_*PGK1*_ *-CAHS3-T*_*PGK1*_	This study
pMAHS	*BY4741 + pRS426-P*_*PGK1*_ *-MAHS-T*_*PGK1*_	This study
pRvLEAM	*BY4741 + pRS426-P*_*PGK1*_ *-RvLEAM-T*_*PGK1*_	This study
pCAHS3-GFP	*BY4741 + pRS426-P*_*PGK1*_ *-CAHS3-AcGFP1-T*_*PGK1*_	This study
pMAHS-GFP	*BY4741 + pRS426-P*_*PGK1*_ *-MAHS-AcGFP1-T*_*PGK1*_	This study
pRvLEAM-GFP	*BY4741 + pRS426-P*_*PGK1*_ *-RvLEAM-AcGFP1-T*_*PGK1*_	This study
pControl	*BY4741 + pRS426-empty*	This study

### Plasmid and strain construction, and plasmid sequencing

pIW14 (pRS426-P_PGK1_-T_PGK1_), a high copy (2μ) plasmid generated in our laboratory previously, was used as a template for the cloning of the tardigrade genes. Q5^®^ High-Fidelity DNA Polymerase (M0491; New England Biolabs, Ipswich, MA, USA) was used for all PCR amplifications. pIW14 was PCR amplified in order to reconstitute the full-length PGK1 promoter and insert the tardigrade genes and/or AcGFP1 by performing a Gibson assembly following the instructions of NEBuilder® HiFi DNA Assembly Master Mix and NEBuilder® Protocol Calculator (New England Biolabs, Ipswich, MA, USA). The inserts were PCR amplified from plasmids obtained from Addgene using the primers shown in Table 2. pAcGFP1-N1-CAHS3 was a gift from Takekazu Kunieda (Addgene plasmid # 90031; http://n2t.net/addgene:90031; RRID: Addgene_90031). pAcGFP1-N1-MAHS was a gift from Takekazu Kunieda (Addgene plasmid # 90034; http://n2t.net/addgene:90034; RRID: Addgene_90034). pAcGFP1-N1-RvLEAM was a gift from Takekazu Kunieda (Addgene plasmid # 90035; http://n2t.net/addgene:90035; RRID: Addgene_90035).

**Table 2:** Primers and plasmids used.

Primers	Description	Sequence
MLL497	pIW14 RV	ttgttgtaaaaagtagataattacttccttgaTGATCTGTAAAAAAGAGAAAAAGAAAGC
MLL498	pIW14-CAHS3 FWD	tgaacgccgataaatATTGAATTGAATTGAAATCGATAGATC
MLL500	pIW14-MAHS FWD	cgagggtgtttaaatATTGAATTGAATTGAAATCGATAGATC
MLL501	pIW14-RvLEAM FWD	cggtcgacgataaatATTGAATTGAATTGAAATCGATAGATC
MLL504	pIW14-AcGFP1 FWD	gagctgtacaagtaaatATTGAATTGAATTGAAATCGATAGATC
MLL505	HisTag-PGK1 FWD	gaagtaattatctactttttacaacaaatataaaacaATGCACCATCACCATCACCATGG
MLL507	CAHS3 RV	aattcaatatTTATCGGCGTTCAGTGTGTTGTTC
MLL510	MAHS+PGK1 FWD	ggaagtaattatctactttttacaacaaatataaaacaATGTCCAGATACCTGCTGCGCG
MLL511	MAHS RV for plasmid	aattcaatatTTAAACACCCTCGTTTTTAACTGCAACTCC
MLL512	RvLEAM+PGK1 FWD	aaggaagtaattatctactttttacaacaaatataaaacaATGTTTCTCGCCCGAAACGC
MLL513	RvLEAM RV for plasmid	aattcaatatTTATCGTCGACCGCCAGCTTG
MLL517	AcGFP1 RV for plasmid	aattcaatatTTACTTGTACAGCTCATCCATGCCG
MLL519	CAHS3 + PGK1 FWD	ggaagtaattatctactttttacaacaaatataaaacaATGTCTTCCCGACAGAACCAGC
MLL530	AcGFP1 FWD + PGK1pr	agtaattatctactttttacaacaaatataaaacaATGGTGAGCAAGGGCGCC
**Plasmid**	**Description**	**Source**
pIW14 (pYPGK)	pRS426 derivative; PGK1p-PGK1t	Loebs et al. [36]
90031	*pAcGFP1-N1-CAHS3*	Addgene
90034	*pAcGFP1-N1-MAHS*	Addgene
90035	*pAcGFP1-N1-RvLEAM*	Addgene
pCRG209	*pRS426-P*_*PGK1*_ *-CAHS3-AcGFP1-T*_*PGK1*_	This study
pCRG211	*pRS426-P*_*PGK1*_ *-MAHS-AcGFP1-T*_*PGK1*_	This study
pCRG212	*pRS426-P*_*PGK1*_ *-RvLEAM-AcGFP1-T*_*PGK1*_	This study
pCRG215	*pRS426-P*_*PGK1*_ *-MAHS-T*_*PGK1*_	This study
pCRG218	*pRS426-P*_*PGK1*_ *-AcGFP1-T*_*PGK1*_	This study
pCRG219	*pRS426-P*_*PGK1*_ *-CAHS3-T*_*PGK1*_	This study
pCRG220	*pRS426-P*_*PGK1*_ *-RvLEAM-T*_*PGK1*_	This study
pCRG243	*pRS426-empty*	This study

The assembly products were transformed into *E. coli* by heat shock at 42ºC for 30s. Plasmids were extracted from *E. coli* with ZR Plasmid Miniprep – Classic (Zymo Research Corp., Irvine, CA, USA). DNA concentration was measured using Biodrop Duo+ (Biochrom US, Holliston, MA, USA). DNA sequences were verified by whole plasmid sequencing performed by Plasmidsaurus (Eugene, OR, USA). Plasmids were transformed into BY4741 using a standard LiAc/SS carrier DNA/PEG protocol [59]. Full sequences of all plasmids are in Supporting Information in S1 File.

### Growth curve

pControl, pCAHS3, pMAHS and pRvLEAM were plated on a SC -Ura plate for 3 days and three colonies of each strain were picked and grown in 5 mL of SC -Ura media overnight, in a shaking incubator (INCU-SHAKER™ 10LR; Benchmark Scientific, Sayreville, NJ, USA) at 30°C and 200 rpm. Then, the OD_600_ of each sample was measured using Biodrop Duo+ (Biochrom US, Holliston, MA, USA)and the cells were diluted to an 0.05 OD_600_ in 6 mL of SC -Ura and 1 mL was used to measure the OD_600_ ending with a 5 mL culture for each strain. The OD_600_ was measured in triplicate for every sample obtaining in this way nine data points for each of the four strains at time 0 h, after 11 h, and measured every hour from the 13 h to the 24 h time point with a last measurement after 35 h to measure the OD_600_ at stationary phase.

### Stress tolerance phenotyping

When performing stress tolerance experiments and controls, yeast cultures were grown at 30°C in a minimal media version of SC -Ura (mSC -Ura) containing 1.7 g/L YNB, 5 g/L Ammonium Sulfate, 0.683 g/L minimal Dropout mix without uracil (0.25 g Adenine, 1 g Histidine, 1 g Inositol, 5 g Leucine, 1 g Methionine, 0.1 g para-Aminobenzoic acid) and 20 g/L glucose, and 2–3% agar when required. For the phenotyping under hyperosmotic stress tolerance mSC -Ura medium contained 5%, 7.5%, 10% or 12% NaCl and 3% agar. For the stress tolerance phenotyping under chronic thermal stress experiments, we used mSC -Ura medium at 37°C and 40°C with 20 g/L glucose. For butanol stress tolerance phenotyping, mSC -Ura medium contained 1%, 1.5%, 2%, 3%, 4% and 5% (v/v) butanol and 2% (w/v) agar. Glucose and NaCl solutions were autoclaved separate from each other and the rest of the media preparations.

Strains containing plasmids were grown overnight at 200 rpm in 14 mL tubes (Fisher Scientific, Waltham, MA, USA) with 5 mL of mSC -Ura media at 30°C starting with a single colony for 2 days until they reached stationary phase. The night before the experiments, 50 μL of the liquid cultures (100 μL of pCAHS3) were sub-cultured into new 14 mL tubes with 5 mL of mSC -Ura having an initial OD_600_ of ~0.1 (OD_600_ of ~0.2 for pCAHS3) until they reached log phase (OD_600_ between 3 and 7). While in log phase, each strain was diluted to a consistent OD_600_ of 0.5 in 500 μL of water in a 1.5mL tube. For the phenotyping under chronic hyperosmotic (NaCl) stress, butanol-induced stress, and chronic thermal stress, 250 μL of each strain solution were transferred to a well on the first column of a 96-well plate, 200 μL of water was added to the wells on columns 2–6. 5-fold dilutions of each strain were performed by pipetting 50 μL out of the first column with a multichannel pipet and adding it to the next column to the right, samples were mixed by pipetting up and down, this was repeated up/down to the sixth column where 50 μL were discarded after mixing the samples, so every well had the same volume of solution (200 μL). Using a pin replicator, the solutions in the 96-well plate were transferred to the required type of plate in triplicate.

For acute stress tolerance phenotyping, the procedure was carried out as above with the following differences. For heat stress tolerance, the 1.5 mL tubes were placed on a heat block (myBlock™ HL Mini Dry Bath with Heated Lid; Benchmark Scientific, Sayreville, NJ, USA) at 30°C (control), 37°C, 48°C and 50°C for 1 h prior to performing the process described for the other stress conditions above. In this case, 250 μL of each strain control cultured at 30°C was transferred to a well on the first column of a 96-well plate, and 250 μL of each strain subjected to each heat stress condition was transferred to the wells on column 7 (one 96-well plate per condition). 200 μL of water was added to the wells on columns 2–6 and 8–12. 5-fold dilutions of each strain were performed as mentioned above for each half of the 96-well plate (columns 1–6 and 7–12). Control plates were grown for 2 days prior to imaging (ChemiDoc MP, Bio-Rad). Other conditions took up to 10 days before imaging. For acute hyperosmotic and desiccation stress, cells were centrifuged at 3000 × g for 5 min and supernatant was discarded, the tubes with the samples for desiccation were left open with 3M Micropore™ Surgical Paper Tape (3M, St. Paul, MN) taped on the aperture and, the cells in the tubes for hyperosmotic stress were resuspended in 1 mL of 15% NaCl mSC -Ura. Both, hyperosmotic and desiccation stress samples were kept at 30ºC in a shaking incubator for 21 h, which were then allowed to recover in mSC -Ura medium for 30 min prior to cell staining and flow cytometry.

### Live/Dead assay and flow cytometry

Plasmid-containing strains were grown overnight as done previously in 14 mL tubes with 5 mL of mSC -Ura media at 200 rpm and 30°C starting at an OD_600_ of 0.1 (and 0.2 for pCAHS3), diluted to a consistent OD_600_ of 0.5 in 1 mL water in 1.5 mL tubes, The tubes were then subjected to a 1-hour incubation at 30°C or 50°C. After the incubation, the tubes were centrifuged at 3000 × g for 5 min to minimize cell death due to centrifuging, and the pellet was resuspended in 1 mL of PBS. Cells were incubated at room temperature (RT) for 30 minutes right after adding 1 μL Live-or-Dye™ 640/662 (CAT# 32007, Biotium, Inc., Fremont, CA, USA) and 1 μL 10 mM Thiazole Orange (CAT# 40077, Biotium, Inc., Fremont, CA, USA) to each tube. The samples were then centrifuged, and the supernatant was discarded. The samples were washed with 1 mL PBS once by centrifuging at 13000 × g for 30 s, resuspended in 500 μL PBS (OD_600_ of 1) and run through a flow cytometer BD Accuri C6 Plus (Becton, Dickinson and Company, Franklin Lakes, NJ, USA) at medium speed (35 μL/min) with the goal of counting 2 x 10^5^ events for each sample, non-fluorescent events were gated out during the flow cytometer run. Cells containing Live-or-Dye were identified as dead when the intensity was higher than 10^4^ using a FL4 filter (661/16 nm; Becton, Dickinson and Company, Franklin Lakes, NJ, USA), everything else was considered to be alive as long as they were identified as fluorescent with an intensity between 10^3^ and 10^5^ using FL1 filter (530/30 nm; Becton, Dickinson and Company, Franklin Lakes, NJ, USA).

### Cell staining and fluorescence microscopy

Strains containing GFP plasmids were grown from a single colony overnight in 14 mL tubes (Fisher Scientific, Waltham, MA, USA) with 5 mL of SC -Ura media at 30°C. The following day, 1 mL of each strain was transferred into a 14 mL culture tube with 4 mL of 2x SC -Ura and 5 μL of 2 mM TMRE and they were grown overnight. The day of imaging, 1 mL of each strain was centrifuged at 3000 × *g* for 5 min and washed once with 0.5 mL of PBS, then the cells were centrifuged at 10000 × *g* for 30 s and the pellet was resuspended in 1 mL SPC buffer [60]. 10 μL of each strain was then transferred onto a glass slide and a 22x22 mm coverslip was placed on top of the droplet. Cell imaging was conducted utilizing an Olympus BX51 microscope equipped with an UPlanFL 100 x 1.30 oil-immersion objective lens and a mercury lamp. Fluorescence micrographs were captured using a Q-Imaging Retiga Exi CCD camera. Processing of the images was carried out employing CellSens Dimension 1.7 software (Olympus America, Center Valley, PA) or Adobe Photoshop. Each experiment involved the analysis of a minimum of three distinct cell populations, with representative images selected for representation, as done previously [61].

### Statistical analysis

For graph making and data analysis, GraphPad Prism version 10.3.0 for Windows (GraphPad Software, Boston, MA, USA) was used. For the statistical analysis of the growth curves, data was fit to a modified Gompertz equation: $\ln\left(\frac{N}{N_{0}}\right)=Ae^{{-e}^{\left(\frac{\mu }{A}e(\lambda -t)+1\right)}}$ [37,38]. For each growth curve, *μ* was determined through least squares fitting (MATLAB 2022a; Mathworks, Natick, MA). One-way ANOVA followed by Dunnett’s multiple comparison test was used to determine growth difference from control (n = 3). For the statistical analysis of the data related to heat shock (n = 3), a Two-way ANOVA followed by Bonferroni’s multiple comparison test was performed. When analyzing the Live/Dead data obtained from the 4 strains under control, hyperosmotic and desiccation conditions; a Two-way ANOVA was performed followed by Dunnett’s multiple comparison test. All data presented in the figures is provided in Supporting Information in S2 File.

## Supporting information

S1 File This file contains Fig S1, Fig S2, and Fig S3 referenced in the text, as well as the full sequences of all plasmids referenced in the text. (PDF)

S2 File This file contains the raw data used to generate figures and run statistics. (XLSX)
