# Supplementary material for: Expressing intrinsically-disordered tardigrade proteins has positive effects on acute but not chronic stress tolerance in Saccharomyces cerevisiae
Source: PLoS One. 2025 Jun 6;20(6):e0325682. doi: 10.1371/journal.pone.0325682 (PMC12143556; doi:10.1371/journal.pone.0325682)
Supplement: S1 File — (PDF) [file pone.0325682.s001.pdf]

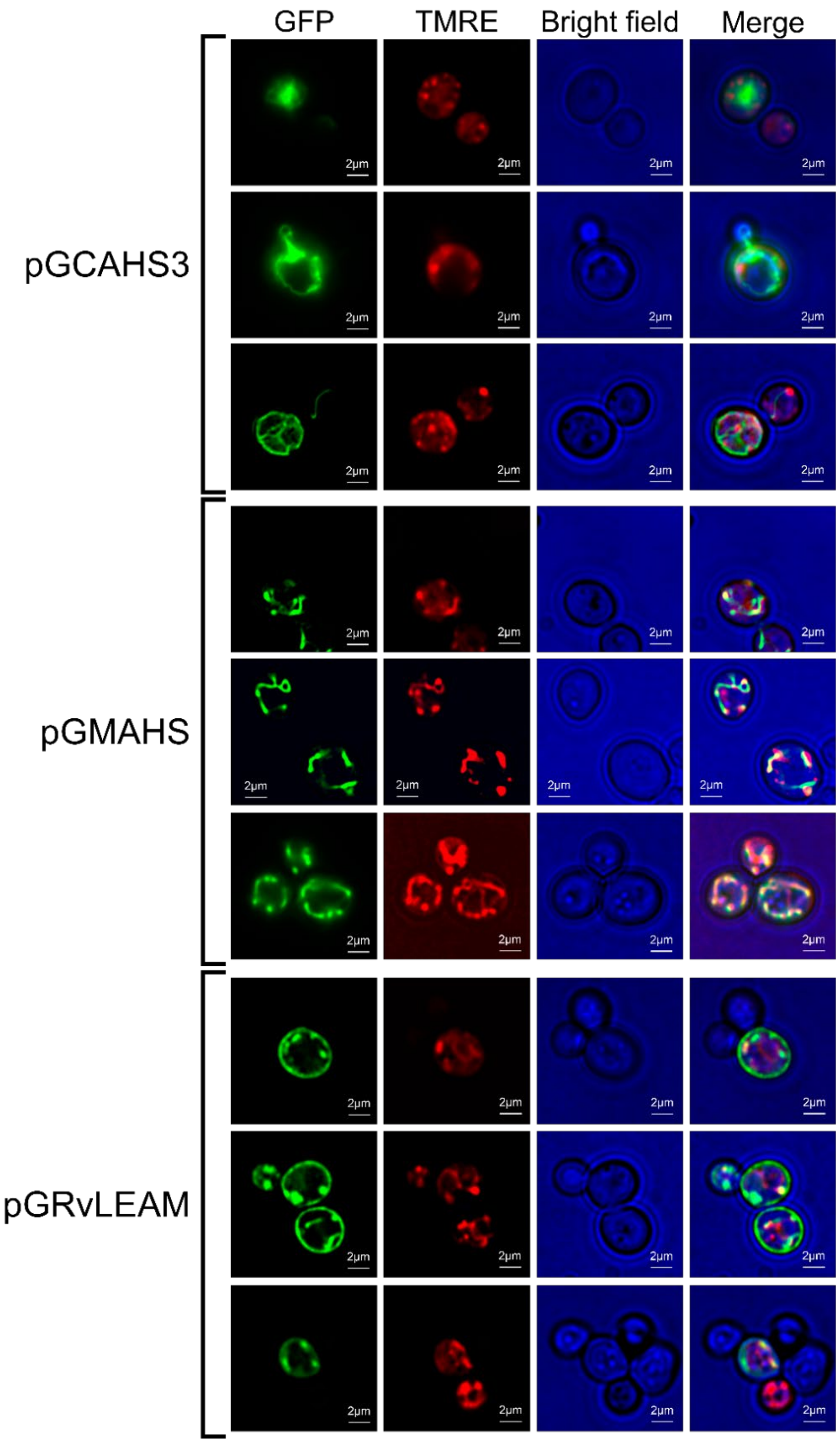

Fig S1. Expression of tardigrade transgenes in S. cerevisiae. Representative fluorescence microscopy images of three populations of S. cerevisiae expressing MAHS-AcGFP1, CAHS3-AcGFP1 and RvLEAM-AcGFP1 (green). Mitochondria are identified with Tetramethylrhodamine ethyl ester perchlorate (TMRE; red) which stains live mitochondria. Images were taken using a 100x objective and merged images of green fluorescence, red fluorescence, and brightfield contrast (blue) are shown. Colocalization of red and green fluorescence shows in yellow.

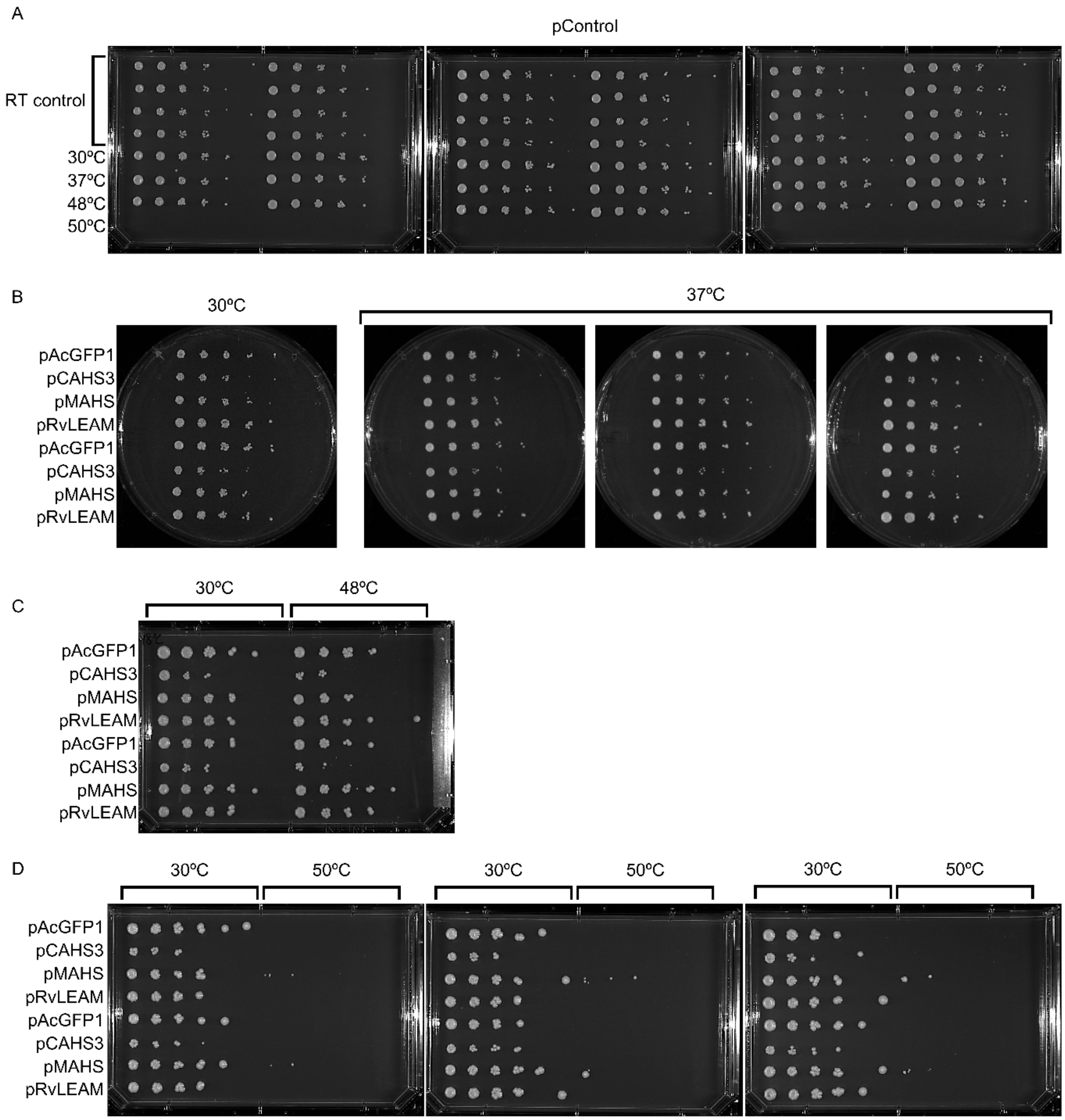

Fig S2. Spot plate growth after heat shock. (A) Images of replicates (n=6) of a 3-day growth of pControl spot-plated in a 5-fold serial dilution starting at an OD_{600} of 0.5 after 1 h heat shock at 30ºC, 37ºC, 48ºC and 50ºC; prior to heat shock samples were split into two 1.5 mL tubes, one of which was kept at room temperature (RT) as a control for each condition. **(B)** Images of replicates (n=6) of the different plasmid-carrying yeast strains spot-plated as in (A) 3 days after a 1 h heat shock at 37ºC on mSC -Ura, 30ºC was used as the heat shock control condition. **(C)** Images of replicates (n=2) of the different spot-plated plasmid-carrying yeast strains 3 days after a 1 h heat shock at 48ºC on mSC -Ura, 30ºC as the heat shock control on the left side of the plate. **(D)** Images of replicates (n=6) of the different spot-plated plasmid-carrying yeast strains 3 days after a 1 h heat shock at 50ºC on mSC -Ura, 30ºC as the control on the left side of the plate.

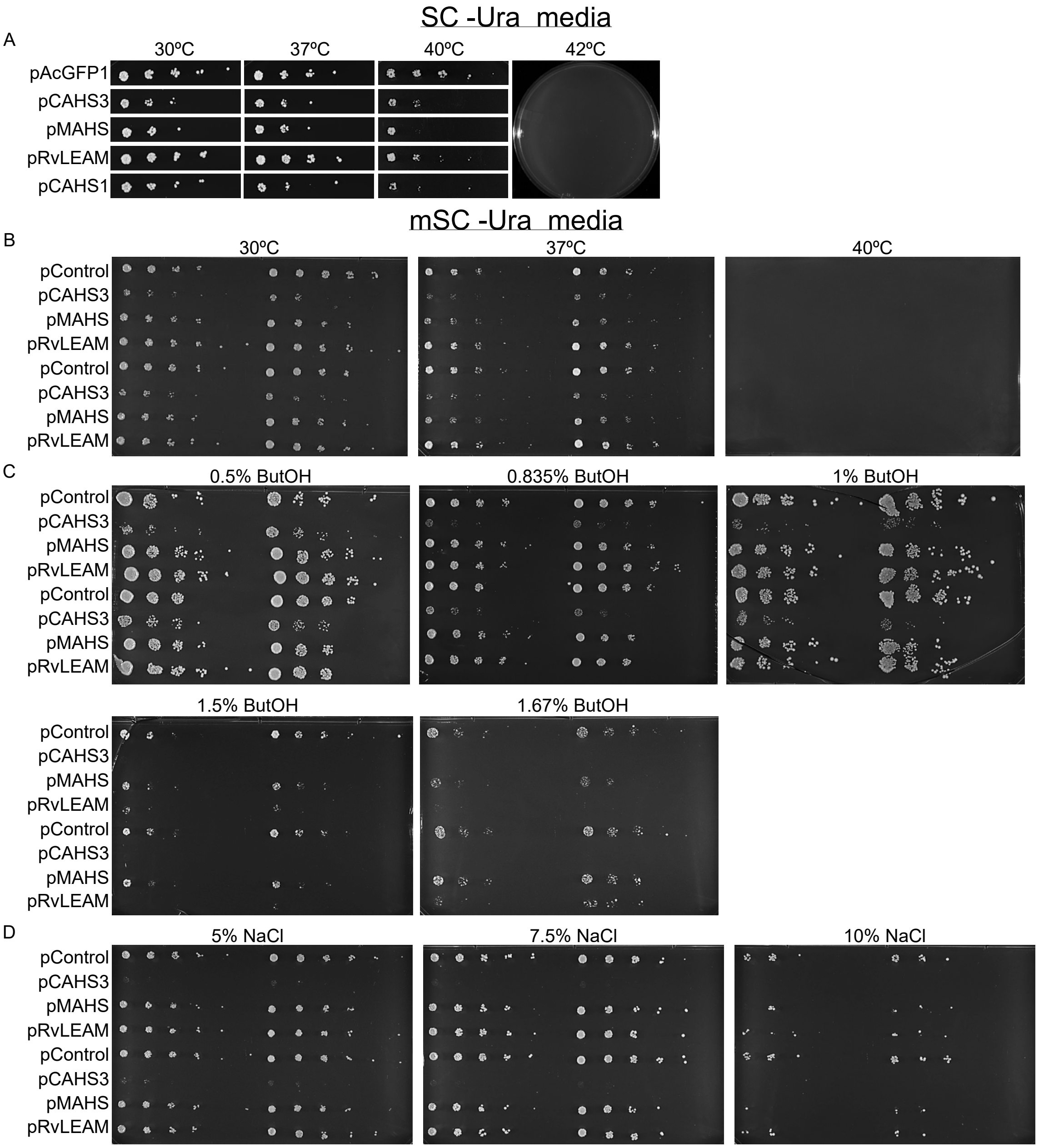

Fig S3. Spot plate growth under chronic stress. (A) Representative images of the different plasmid-carrying yeast strains spot-plated in a 5-fold serial dilution starting at an OD_{600} of 0.5 after 2 days of growth at 30ºC and 37ºC, 3 days at 40ºC and 5 days at 42ºC on SC -Ura. **(B)** Images of replicates (n=4) of the different plasmid-carrying yeast strains spot-plated as in (A) after 2 days of growth at 30ºC and 37ºC, and 10 days at 40ºC on mSC -Ura. **(C)** Images of spot-plated replicates (n=4) of the plasmid-carrying yeast strains after 3 days of growth in 0.5% and 0.835% ButOH, after 5 days of growth in 1% ButOH and, after 8 days of growth at 1.5% and 1.67% ButOH. **(D)** Images of the spot-plated yeast replicates (n=4) after 3 days in 5% NaCl, after 6 days in 7.5% NaCl and after 9 days of growth in 10% NaCl.

The Full Sequences of Plasmids Constructed in This Study

pControl

5’-
aattgaagctctaatttgtgagtttagtatacatgcatttacttataatacagttttttagttttgctggccgcatcttctcaaatatgcttcccagcct
gcttttctgtaacgttcaccctctaccttagcatcccttccctttgcaaatagtcctcttccaacaataataatgtcagatcctgtagagaccacatc
atccacggttctatactgttgacccaatgcgtctcccttgtcatctaaacccacaccgggtgtcataatcaaccaatcgtaaccttcatctcttcca
cccatgtctctttgagcaataaagccgataacaaaatctttgtcgctcttcgcaatgtcaacagtacccttagtatattctccagtagatagggag
cccttgcatgacaattctgctaacatcaaaaggcctctaggttcctttgttacttcttctgccgcctgcttcaaaccgctaacaatacctgggccca
ccacaccgtgtgcattcgtaatgtctgcccattctgctattctgtatacacccgcagagtactgcaatttgactgtattaccaatgtcagcaaatttt
ctgtcttcgaagagtaaaaaattgtacttggcggataatgcctttagcggcttaactgtgccctccatggaaaaatcagtcaagatatccacatg
tgtttttagtaaacaaattttgggacctaatgcttcaactaactccagtaattccttggtggtacgaacatccaatgaagcacacaagtttgtttgc
ttttcgtgcatgatattaaatagcttggcagcaacaggactaggatgagtagcagcacgttccttatatgtagctttcgacatgatttatcttcgttt
cctgcaggtttttgttctgtgcagttgggttaagaatactgggcaatttcatgtttcttcaacactacatatgcgtatatataccaatctaagtctgtg
ctccttccttcgttcttccttctgttcggagattaccgaatcaaaaaaatttcaaggaaaccgaaatcaaaaaaaagaataaaaaaaaaatgat
gaattgaaaaggtggtatggtgcactctcagtacaatctgctctgatgccgcatagttaagccagccccgacacccgccaacacccgctgacgc
gccctgacgggcttgtctgctcccggcatccgcttacagacaagctgtgaccgtctccgggagctgcatgtgtcagaggttttcaccgtcatcacc
gaaacgcgcgagacgaaagggcctcgtgatacgcctatttttataggttaatgtcatgataataatggtttcttagtatgatccaatatcaaagg
aaatgatagcattgaaggatgagactaatccaattgaggagtggcagcatatagaacagctaaagggtagtgctgaaggaagcatacgatac
cccgcatggaatgggataatatcacaggaggtactagactacctttcatcctacataaatagacgcatataagtacgcatttaagcataaacac
gcactatgccgttcttctcatgtatatatatatacaggcaacacgcagatataggtgcgacgtgaacagtgagctgtatgtgcgcagctcgcgtt
gcattttcggaagcgctcgttttcggaaacgctttgaagttcctattccgaagttcctattctctagaaagtataggaacttcagagcgcttttgaaa
accaaaagcgctctgaagacgcactttcaaaaaaccaaaaacgcaccggactgtaacgagctactaaaatattgcgaataccgcttccacaa
acattgctcaaaagtatctctttgctatatatctctgtgctatatccctatataacctacccatccacctttcgctccttgaacttgcatctaaactcg
acctctacattttttatgtttatctctagtattactctttagacaaaaaaattgtagtaagaactattcatagagtgaatcgaaaacaatacgaaaa
tgtaaacatttcctatacgtagtatatagagacaaaatagaagaaaccgttcataatttctgaccaatgaagaatcatcaacgctatcactttct
gttcacaaagtatgcgcaatccacatcggtatagaatataatcggggatgcctttatcttgaaaaaatgcacccgcagcttcgctagtaatcagt
aaacgcgggaagtggagtcaggctttttttatggaagagaaaatagacaccaaagtagccttcttctaaccttaacggacctacagtgcaaaa
agttatcaagagactgcattatagagcgcacaaaggagaaaaaagtaatctaagatgctttgttagaaaaatagcgctctcgggatgcatttt
tgtagaacaaaaaagaagtatagattctttgttggtaaaatagcgctctcgcgttgcatttctgttctgtaaaaatgcagctcagattctttgtttga
aaaattagcgctctcgcgttgcattttgttttacaaaaatgaagcacagattcttcgttggtaaaatagcgctttcgcgttgcatttctgttctgtaa
aaatgcagctcagattctttgtttgaaaaattagcgctctcgcgttgcattttgttctacaaaatgaagcacagatgcttcgttcaggtggcacttt
tcggggaaatgtgcgcggaacccctatttgtttatttttctaaatacattcaaatatgtatccgctcatgagacaataaccctgataaatgcttcaa
taatattgaaaaaggaagagtatgagtattcaacatttccgtgtcgcccttattcccttttttgcggcattttgccttcctgtttttgctcacccagaa
acgctggtgaaagtaaaagatgctgaagatcagttgggtgcacgagtgggttacatcgaactggatctcaacagcggtaagatccttgagagt
tttcgccccgaagaacgttttccaatgatgagcacttttaaagttctgctatgtggcgcggtattatcccgtattgacgccgggcaagagcaactc
ggtcgccgcatacactattctcagaatgacttggttgagtactcaccagtcacagaaaagcatcttacggatggcatgacagtaagagaattat
gcagtgctgccataaccatgagtgataacactgcggccaacttacttctgacaacgatcggaggaccgaaggagctaaccgcttttttgcacaa
catgggggatcatgtaactcgccttgatcgttgggaaccggagctgaatgaagccataccaaacgacgagcgtgacaccacgatgcctgtagc
aatggcaacaacgttgcgcaaactattaactggcgaactacttactctagcttcccggcaacaattaatagactggatggaggcggataaagtt
gcaggaccacttctgcgctcggcccttccggctggctggtttattgctgataaatctggagccggtgagcgtgggtctcgcggtatcattgcagca
ctggggccagatggtaagccctcccgtatcgtagttatctacacgacggggagtcaggcaactatggatgaacgaaatagacagatcgctgag
ataggtgcctcactgattaagcattggtaactgtcagaccaagtttactcatatatactttagattgatttaaaacttcatttttaatttaaaaggat
ctaggtgaagatcctttttgataatctcatgaccaaaatcccttaacgtgagttttcgttccactgagcgtcagaccccgtagaaaagatcaaagg
atcttcttgagatcctttttttctgcgcgtaatctgctgcttgcaaacaaaaaaaccaccgctaccagcggtggtttgtttgccggatcaagagcta
ccaactctttttccgaaggtaactggcttcagcagagcgcagataccaaatactgttcttctagtgtagccgtagttaggccaccacttcaagaac

tctgtagcaccgcctacatacctcgctctgctaatcctgttaccagtggctgctgccagtggcgataagtcgtgtcttaccgggttggactcaaga
cgatagttaccggataaggcgcagcggtcgggctgaacggggggttcgtgcacacagcccagcttggagcgaacgacctacaccgaactgag
atacctacagcgtgagctatgagaaagcgccacgcttcccgaagggagaaaggcggacaggtatccggtaagcggcagggtcggaacagg
agagcgcacgagggagcttccagggggaaacgcctggtatctttatagtcctgtcgggtttcgccacctctgacttgagcgtcgatttttgtgatg
ctcgtcaggggggcggagcctatggaaaaacgccagcaacgcggcctttttacggttcctggccttttgctggccttttgctcacatgttctttcct
gcgttatcccctgattctgtggataaccgtattaccgcctttgagtgagctgataccgctcgccgcagccgaacgaccgagcgcagcgagtcagt
gagcgaggaagcggaagagcgcccaatacgcaaaccgcctctccccgcgcgttggccgattcattaatgcagctggcacgacaggtttcccga
ctggaaagcgggcagtgagcgcaacgcaattaatgtgagttagctcactcattaggcaccccaggctttacactttatgcttccggctcgtatgtt
gtgtggaattgtgagcggataacaatttcacacaggaaacagctatgaccatgattacgccaagcgcgcaattaaccctcactaaagggaaca
aaagctggagctcagccacaatagaagctttctaactgatctatccaaaactgaaaattacattcttgattaggtttatcacaggcaaatgt-3’

pacGFP1

5’-
acgaaagggcctcgtgatacgcctatttttataggttaatgtcatgataataatggtttcttagtatgatccaatatcaaaggaaatgatagcatt
gaaggatgagactaatccaattgaggagtggcagcatatagaacagctaaagggtagtgctgaaggaagcatacgataccccgcatggaat
gggataatatcacaggaggtactagactacctttcatcctacataaatagacgcatataagtacgcatttaagcataaacacgcactatgccgtt
cttctcatgtatatatatatacaggcaacacgcagatataggtgcgacgtgaacagtgagctgtatgtgcgcagctcgcgttgcattttcggaag
cgctcgttttcggaaacgctttgaagttcctattccgaagttcctattctctagaaagtataggaacttcagagcgcttttgaaaaccaaaagcgct
ctgaagacgcactttcaaaaaaccaaaaacgcaccggactgtaacgagctactaaaatattgcgaataccgcttccacaaacattgctcaaaa
gtatctctttgctatatatctctgtgctatatccctatataacctacccatccacctttcgctccttgaacttgcatctaaactcgacctctacattttt
atgtttatctctagtattactctttagacaaaaaaattgtagtaagaactattcatagagtgaatcgaaaacaatacgaaaatgtaaacatttcct
atacgtagtatatagagacaaaatagaagaaaccgttcataattttctgaccaatgaagaatcatcaacgctatcactttctgttcacaaagtat
gcgcaatccacatcggtatagaatataatcggggatgcctttatcttgaaaaaatgcacccgcagcttcgctagtaatcagtaaacgcgggaag
tggagtcaggctttttttatggaagagaaaatagacaccaaagtagccttcttctaaccttaacggacctacagtgcaaaaagttatcaagaga
ctgcattatagagcgcacaaaggagaaaaaaagtaatctaagatgctttgttagaaaaatagcgctctcgggatgcatttttgtagaacaaaa
aagaagtatagattctttgttggtaaaatagcgctctcgcgttgcatttctgttctgtaaaaatgcagctcagattctttgtttgaaaaattagcgct
ctcgcgttgcattttgttttacaaaaatgaagcacagattcttcgttggtaaaatagcgctttcgcgttgcatttctgttctgtaaaaatgcagctca
gattctttgtttgaaaaattagcgctctcgcgttgcattttgttctacaaaatgaagcacagatgcttcgttcaggtggcacttttcggggaaatgt
gcgcggaacccctatttgtttatttttctaaatacattcaaatatgtatccgctcatgagacaataaccctgataaatgcttcaataatattgaaaa
aggaagagtatgagtattcaacatttccgtgtcgcccttattcccttttttgcggcattttgccttcctgtttttgctcacccagaaacgctggtgaaa
gtaaaagatgctgaagatcagttgggtgcacgagtgggttacatcgaactggatctcaacagcggtaagatccttgagagttttcgccccgaag
aacgttttccaatgatgagcacttttaaagttctgctatgtggcgcggtattatcccgtattgacgccgggcaagagcaactcggtcgccgcatac
actattctcagaatgacttggttgagtactcaccagtcacagaaaagcatcttacggatggcatgacagtaagagaattatgcagtgctgccata
accatgagtgataacactgcggccaacttacttctgacaacgatcggaggaccgaaggagctaaccgcttttttgcacaacatgggggatcatg
taactcgccttgatcgttgggaaccggagctgaatgaagccataccaaacgacgagcgtgacaccacgatgcctgtagcaatggcaacaacgt
tgcgcaaactattaactggcgaactacttactctagcttcccggcaacaattaatagactggatggaggcggataaagttgcaggaccacttct
gcgctcggcccttccggctggctggtttattgctgataaatctggagccggtgagcgtgggtctcgcggtatcattgcagcactggggccagatg
gtaagccctcccgtatcgtagttatctacacgacggggagtcaggcaactatggatgaacgaaatagacagatcgctgagataggtgcctcact
gattaagcattggtaactgtcagaccaagtttactcatatatactttagattgatttaaaacttcatttttaatttaaaaggatctaggtgaagatcc
tttttgataatctcatgaccaaaatcccttaacgtgagttttcgttccactgagcgtcagaccccgtagaaaagatcaaaggatcttcttgagatcc
tttttttctgcgcgtaatctgctgcttgcaaacaaaaaaaccaccgctaccagcggtggtttgtttgccggatcaagagctaccaactctttttccg
aaggtaactggcttcagcagagcgcagataccaaatactgtTcttctagtgtagccgtagttaggccaccacttcaagaactctgtagcaccgc
ctacatacctcgctctgctaatcctgttaccagtggctgctgccagtggcgataagtcgtgtcttaccgggttggactcaagacgatagttaccgg
ataaggcgcagcggtcgggctgaacggggggttcgtgcacacagcccagcttggagcgaacgacctacaccgaactgagatacctacagcgt
gagctatgagaaagcgccacgcttcccgaagggagaaaggcggacaggtatccggtaagcggcagggtcggaacaggagagcgcacgag
ggagcttccagggggaaacgcctggtatctttatagtcctgtcgggtttcgccacctctgacttgagcgtcgatttttgtgatgctcgtcaggggg

gcggagcctatggaaaaacgccagcaacgcggcctttttacggttcctggccttttgctggccttttgctcacatgttctttcctgcgttatcccctg
attctgtggataaccgtattaccgcctttgagtgagctgataccgctcgccgcagccgaacgaccgagcgcagcgagtcagtgagcgaggaag
cggaagagcgcccaatacgcaaaccgcctctccccgcgcgttggccgattcattaatgcagctggcacgacaggtttcccgactggaaagcgg
gcagtgagcgcaacgcaattaatgtgagttagctcactcattaggcaccccaggctttacactttatgcttccggctcgtatgttgtgtggaattgt
gagcggataacaatttcacacaggaaacagctatgaccatgattacgccaagcgcgcaattaaccctcactaaagggaacaaaagctggAG
CTCAgccacaatagaagctttctaactgatctatccaaaactgaaaattacattcttgattaggtttatcacaggcaaatgtaatttgtggtatttt
gccgttcaaaatctgtagaattttctcattggtcacattacaacctgaaaatactttatctacaatcataccattcttataacatgtccccttaatact
aggatcaggcatgaacgcatcacagacaaaatcttcttgacaaacgtcacaattgatccctccccatccgttatcacaatgacaggtgtcatttt
gtgctcttatgggacgatccttattaccgctttcatccggtgatagaccgccacagaggggcagagagcaatcatcacctgcaaacccttctata
cactcacatctaccagtgtacgaattgcattcagaaaactgtttgcattcaaaaataggtagcatacaattaaaacatggcgggcatgtatcatt
gcccttatcttgtgcagttagacgcgaattttcgaagaagtaccttcaaagaatggggtcttatcttgttttgcaagtaccactgagcaggataat
aatagaaatgataatatactatagtagagataacgtcgatgacttcccatactgtaattgcttttagttgtgtatttttagtgtgcaagtttctgtaa
atcgattaatttttttttctttcctcttttattaaccttaattttattttagattcctgacttcaactcaagacgcacagatattataacatctgcataat
aggcatttgcaagaattactcGTGAGTAAGGAAAGAGTGAGGAACTATCGCATACCTGCATTTAAAGATGCCGATTTG
GGCGCGAATCCTTTATTTTGGCTTCACCCTCATACTATTATCAGGGCCAGAAAAAGGAAGTGTTTCCCTCCTTC
TTGAATTGATGTTACCCTCATAAAGCACGTGGCCTCTTATCGAGAAAGAAATTACCGTCGCTCGTGATTTGTTT
GCAAAAAGAACAAAACTGAAAAAACCCAGACACGCTCGACTTCCTGTCTTCCTATTGATTGCAGCTTCCAATT
TCGTCACACAACAAGGTCCTAGCGACGGCTCACAGGTTTTGTAACAAGCAATCGAAGGTTCTGGAATGGCGG
GAAAGGGTTTAGTACCACATGCTATGATGCCCACTGTGATCTCCAGAGCAAAGTTCGTTCGATCGTACTGTTAC
TCTCTCTCTTTCAAACAGAATTGTCCGAATCGTGTGACAACAACAGCCTGTTCTCACACACTCTTTTCTTCTAAC
CAAGGGGGTGGTTTAGTTTAGTAGAACCTCGTGAAACTTACATTTACATATATATAAACTTGCATAAATTGGTCA
ATGCAAGAAATACATATTTGGTCTTTTCTAATTCGTAGTTTTTCAAGTTCTTAGATGCTTTCTTTTTCTCTTTTTTA
CAGATCATCAAGGAAGTAATTATCTACTTTTTACAACAAATATAAAACAatggtgagcaagggcgccgagctgttcaccgg
catcgtgcccatcctgatcgagctgaatggcgatgtgaatggccacaagttcagcgtgagcggcgagggcgagggcgatgccacctacggca
agctgaccctgaagttcatctgcaccaccggcaagctgcctgtgccctggcccaccctggtgaccaccctgagctacggcgtgcagtgcttctca
cgctaccccgatcacatgaagcagcacgacttcttcaagagcgccatgcctgagggctacatccaggagcgcaccatcttcttcgaggatgacg
gcaactacaagtcgcgcgccgaggtgaagttcgagggcgataccctggtgaatcgcatcgagctgaccggcaccgatttcaaggaggatggc
aacatcctgggcaataagatggagtacaactacaacgcccacaatgtgtacatcatgaccgacaaggccaagaatggcatcaaggtgaactt
caagatccgccacaacatcgaggatggcagcgtgcagctggccgaccactaccagcagaatacccccatcggcgatggccctgtgctgctgc
ccgataaccactacctgtccacccagagcgccctgtccaaggaccccaacgagaagcgcgatcacatgatctacttcggcttcgtgaccgccgc
cgccatcacccacggcatggatgagctgtacaagtaaATATTGAATTGAATTGAAATCGATAGATCAATTTTTTTCTTTTCTC
TTTCCCCATCCTTTACGCTAAAATAATAGTTTATTTTATTTTTTGAATATTTTTTATTTATATACGTATATATAGACTA
TTATTTATCTTTTAATGATTATTAAGATTTTTATTAAAAAAAAATTCGCTCCTCTTTTAATGCCTTTATGCAGTTTTT
TTTTCCCATTCGATATTTCTATGTTCGGGTTCAGCGTATTTTAAGTTTAATAACTCGAAAATTCTGCGTTCGTTAA
AGCTTTCGAGAAGGATATTATTTCGAAATAAACCGTGTTGTGTAAGCTTGAAGCCTTTTTGCGCTGCCAATATT
CTTATCCATCTATTGTACTCTTTAGATCCAGTATAGTGTATTCTTCCTGCTCCAAGCTCATCCCACTTGCAACAAA
AAAAGTCTAATCTTCTGCAATAATTTCCATCCTTGGCATTCAGAGACATATATTGGTCAATCGGTTTTAATTTGgta
cccaattcgccctatagtgagtcgtattacgcgcgctcactggccgtcgttttacaacgtcgtgactgggaaaaccctggcgttacccaacttaat
cgccttgcagcacatccccctttcgccagctggcgtaatagcgaagaggcccgcaccgatcgcccttcccaacagttgcgcagcctgaatggcg
aatggacgcgccctgtagcggcgcattaagcgcggcgggtgtggtggttacgcgcagcgtgaccgctacacttgccagcgccctagcgcccgc
tcctttcgctttcttcccttcctttctcgccacgttcgccggctttccccgtcaagctctaaatcgggggctccctttagggttccgatttagtgctttac
ggcacctcgaccccaaaaaacttgattagggtgatggttcacgtagtgggccatcgccctgatagacggtttttcgccctttgacgttggagtcca
cgttctttaatagtggactcttgttccaaactggaacaacactcaaccctatctcggtctattcttttgatttataagggattttgccgatttcggcct
attggttaaaaaatgagctgatttaacaaaaatttaacgcgaattttaacaaaatattaacgCttacaatttcctgatgcggtattttctccttacg
catctgtgcggtatttcacaccgcatagggtaataactgatataattaaattgaagctctaatttgtgagtttagtatacatgcatttacttataata
cagtttttagttttgctggccgcatcttctcaaatatgcttcccagcctgcttttctgtaacgttcaccctctaccttagcatcccttccctttgcaaat
agtcctcttccaacaataataatgtcagatcctgtagagaccacatcatccacggttctatactgttgacccaatgcgtctcccttgtcatctaaac
ccacaccgggtgtcataatcaaccaatcgtaaccttcatctcttccacccatgtctctttgagcaataaagccgataacaaaatctttgtcgctctt

cgcaatgtcaacagtacccttagtatattctccagtagatagggagcccttgcatgacaattctgctaacatcaaaaggcctctaggttcctttgtt
acttcttctgccgcctgcttcaaaccgctaacaatacctgggcccaccacaccgtgtgcattcgtaatgtctgcccattctgctattctgtatacac
ccgcagagtactgcaatttgactgtattaccaatgtcagcaaattttctgtcttcgaagagtaaaaaattgtacttggcggataatgcctttagcg
gcttaactgtgccctccatggaaaaatcagtcaagatatccacatgtgtttttagtaaacaaattttgggacctaatgcttcaactaactccagta
attccttggtggtacgaacatccaatgaagcacacaagtttgtttgcttttcgtgcatgatattaaatagcttggcagcaacaggactaggatgag
tagcagcacgttccttatatgtagctttcgacatgatttatcttcgtttcctgcaggtttttgttctgtgcagttgggttaagaatactgggcaatttca
tgtttcttcaacactacatatgcgtatatataccaatctaagtctgtgctccttccttcgttcttccttctgttcggagattaccgaatcaaaaaaattt
caaGgaaaccgaaatcaaaaaaaaagaataaaaaaaaaaatgatgaattgaaaaggtggtatggtgcactctcagtacaatctgctctgatgc
cgcatagttaagccagccccgacacccgccaacacccgctgacgcgccctgacgggcttgtctgctcccggcatccgcttacagacaagctgtg
accgtctccgggagctgcatgtgtcagaggttttcaccgtcatcaccgaaacgcgcgag-3’

pCAHS3

5’-
acgaaagggcctcgtgatacgcctatttttataggttaatgtcatgataataatggtttcttagtatgatccaatatcaaaggaaatgatagcatt
gaaggatgagactaatccaattgaggagtggcagcatatagaacagctaaagggtagtgctgaaggaagcatacgataccccgcatggaat
gggataatatcacaggaggtactagactacctttcatcctacataaatagacgcatataagtacgcatttaagcataaacacgcactatgccgtt
cttctcatgtatatatatatacaggcaacacgcagatataggtgcgacgtgaacagtgagctgtatgtgcgcagctcgcgttgcattttcggaag
cgctcgttttcggaaacgctttgaagttcctattccgaagttcctattctctagaaagtataggaacttcagagcgcttttgaaaaccaaaagcgct
ctgaagacgcactttcaaaaaaccaaaaacgcaccggactgtaacgagctactaaaatattgcgaataccgcttccacaaacattgctcaaaa
gtatctctttgctatatatctctgtgctatatccctatataacctacccatccacctttcgctccttgaacttgcatctaaactcgacctctacattttt
atgtttatctctagtattactctttagacaaaaaaattgtagtaagaactattcatagagtgaatcgaaaacaatacgaaaatgtaaacatttcct
atacgtagtatatagagacaaaatagaagaaaccgttcataatttctgaccaatgaagaatcatcaacgctatcactttctgttcacaaagtat
gcgcaatccacatcggtatagaatataatcggggatgcctttatcttgaaaaaatgcacccgcagcttcgctagtaatcagtaaacgcgggaag
tggagtcaggctttttttatggaagagaaaatagacaccaaagtagccttcttctaaccttaacggacctacagtgcaaaaagttatcaagaga
ctgcattatagagcgcacaaaggagaaaaaaagtaatctaagatgctttgttagaaaaatagcgctctcgggatgcatttttgtagaacaaaa
aagaagtatagattctttgttggtaaaatagcgctctcgcgttgcatttctgttctgtaaaaatgcagctcagattctttgtttgaaaaattagcgct
ctcgcgttgcatttttgttttacaaaaatgaagcacagattcttcgttggtaaaatagcgctttcgcgttgcatttctgttctgtaaaaatgcagctca
gattctttgtttgaaaaattagcgctctcgcgttgcatttttgttctacaaaatgaagcacagatgcttcgttcaggtggcacttttcggggaaatgt
gcgcggaacccctatttgtttatttttctaaatacattcaaatatgtatccgctcatgagacaataaccctgataaatgcttcaataatattgaaaa
aggaagagtatgagtattcaacatttccgtgtcgcccttattcccttttttgcggcattttgccttcctgtttttgctcacccagaaacgctggtgaaa
gtaaaagatgctgaagatcagttgggtgcacgagtgggttacatcgaactggatctcaacagcggtaagatccttgagagttttcgccccgaag
aacgttttccaatgatgagcacttttaaagttctgctatgtggcgcggtattatcccgtattgacgccgggcaagagcaactcggtcgccgcatac
actattctcagaatgacttggttgagtactcaccagtcacagaaaagcatcttacggatggcatgacagtaagagaattatgcagtgctgccata
accatgagtgataacactgcggccaacttacttctgacaacgatcggaggaccgaaggagctaaccgcttttttgcacaacatgggggatcatg
taactcgccttgatcgttgggaaccggagctgaatgaagccataccaaacgacgagcgtgacaccacgatgcctgtagcaatggcaacaacgt
tgcgcaaactattaactggcgaactacttactctagcttcccggcaacaattaatagactggatggaggcggataaagttgcaggaccacttct
gcgctcggcccttccggctggctggtttattgctgataaatctggagccggtgagcgtgggtctcgcggtatcattgcagcactggggccagatg
gtaagccctcccgtatcgtagttatctacacgacggggagtcaggcaactatggatgaacgaaatagacagatcgctgagataggtgcctcact
gattaagcattggtaactgtcagaccaagtttactcatatatactttagattgatttaaaacttcatttttaatttaaaaggatctaggtgaagatcc
tttttgataatctcatgaccaaaatcccttaacgtgagttttcgttccactgagcgtcagaccccgtagaaaagatcaaaggatcttcttgagatcc
tttttttctgcgcgtaatctgctgcttgcaaacaaaaaaaccaccgctaccagcggtggtttgtttgccggatcaagagctaccaactctttttccg
aaggtaactggcttcagcagagcgcagataccaaatactgttcttctagtgtagccgtagttaggccaccacttcaagaactctgtagcaccgcc
tacatacctcgctctgctaatcctgttaccagtggctgctgccagtggcgataagtcgtgtcttaccgggttggactcaagacgatagttaccgga
taaggcgcagcggtcgggctgaacggggggttcgtgcacacagcccagcttggagcgaacgacctacaccgaactgagatacctacagcgtg
agctatgagaaagcgccacgcttcccgaagggagaaaggcggacaggtatccggtaagcggcagggtcggaacaggagagcgcacgagg
gagcttccagggggaaacgcctggtatctttatagtcctgtcgggtttcgccacctctgacttgagcgtcgatttttgtgatgctcgtcaggggggc

ggagcctatggaaaaacgccagcaacgcggcctttttacggttcctggccttttgctggccttttgctcacatgttctttcctgcgttatcccctgatt
ctgtggataaccgtattaccgcctttgagtgagctgataccgctcgccgcagccgaacgaccgagcgcagcgagtcagtgagcgaggaagcgg
aagagcgcccaatacgcaaaccgcctctccccgcgcgttggccgattcattaatgcagctggcacgacaggtttcccgactggaaagcgggca
gtgagcgcaacgcaattaatgtgagttagctcactcattaggcaccccaggctttacactttatgcttccggctcgtatgttgtgtggaattgtgag
cggataacaatttcacacaggaaacagctatgaccatgattacgccaagcgcgcaattaaccctcactaaagggaacaaaagctggAGCTC
Agccacaatagaagctttctaactgatctatccaaaactgaaaattacattcttgattaggtttatcacaggcaaatgtaatttgtggtattttgcc
gttcaaaatctgtagaattttctcattggtcacattacaacctgaaaatactttatctacaatcataccattcttataacatgtccccttaatactag
gatcaggcatgaacgcatcacagacaaaatcttcttgacaaacgtcacaattgatccctccccatccgttatcacaatgacaggtgtcattttgtg
ctcttatgggacgatccttattaccgctttcatccggtgatagaccgccacagaggggcagagagcaatcatcacctgcaaacccttctatacac
tcacatctaccagtgtacgaattgcattcagaaaactgtttgcattcaaaaataggtagcatacaattaaaacatggcgggcatgtatcattgcc
cttatcttgtgcagttagacgcgaattttcgaagaagtaccttcaaagaatggggtcttatcttgttttgcaagtaccactgagcaggataataat
agaaatgataatatactatagtagagataacgtcgatgacttcccatactgtaattgcttttagttgtgtatttttagtgtgcaagtttctgtaaatc
gattaatttttttttctttcctctttttattaaccttaattttattttagattcctgacttcaactcaagacgcacagatattataacatctgcataatag
gcatttgcaagaattactcgtgagtaaggaaagagtgaggaactatcgcatacctgcatttaaagatgccgatttgggcgcgaatcctttattttg
gcttcaccctcatactattatcagggccagaaaaaggaagtgtttccctccttcttgaattgatgttaccctcataaagcacgtggcctcttatcga
gaaagaaattaccgtcgctcgtgatttgtttgcaaaaagaacaaaactgaaaaaacccagacacgctcgacttcctgtcttcctattgattgcag
cttccaatttcgtcacacaacaaggtcctagcgacggctcacaggttttgtaacaagcaatcgaaggttctggaatggcgggaaagggtttagt
accacatgctatgatgcccactgtgatctccagagcaaagttcgttcgatcgtactgttactctctctctttcaaacagaattgtccgaatcgtgtg
acaacaacagcctgttctcacacactcttttcttctaaccaagggggtggtttagtttagtagaacctcgtgaaacttacatttacatatatataaa
cttgcataaattggtcaatgcaagaaatacatatttggtcttttctaattcgtagtttttcaagttcttagatgctttctttttctctttttttacagatcaT
CAAGGAAGTAATTATCTACTTTTTACAACAAATATAAAACAatgcaccatcaccatcaccatggatccatgtcttcccgacaga
accagcaatcgtccagccaacactcgtcctccagccagcaaggtggtcaaggtggtcaaggtgttcaaggaagttccagctactcgcgcaccg
aggtccacaccagcagtggaggacctaccatcggtggagcccagcgaactgtccccgtccccccctggatctcactccgaggtccatgaggagc
gtgaggtcatcaagcatggtaccaaaaccgaaagcgagacccacgtcgtcaccgtcccagtgacaactttcggcagcaccaacatggaatctg
tccgaaccggcttcaccgtcacccaagacaagaacttgaccgttgctgctcccaacatcgctgctcccatccacagcaacctcgaccttaacctc
ggcggtggagctcgcgctgaaattaccgcagggaccaccgttgacttgagcaagatccagcgcaaggatttgggacctgaagagtatgctcgc
tacaaggccaaggtcgagcaactggccaggcaagatgagcaagacgcgggtatgcgcgctgcccagtaccgagaggaagtggagcgtgat
gccgaactcatccgacagatcttggagcgacaacacatccgtgatcttgaattccgcaaggaaatggttgagaaccaagtcaaccgacaagag
agagaaatccagctggaggctgagtacgcaatgcgagccctcgagcttgagcgcaatgccgctaaagaggctttggaaagcgccaaggccc
agactaacgtcaatgtcaaggtcgagtccgctattggtaccaccgtctccaagggtgcaatccagacttccgccgacaagagcagcaccacca
agactggacccaccaccgtcactcagattaaacataccgaacaacacactgaacgccgataaATATTGAATTGAATTGAAATCGATA
GATCAATTTTTTTCTTTTCTCTTTCCCCATCCTTTACGCTAAAATAATAGTTTATTTTATTTTTTGAATATTTTTTATT
TATATACGTATATATAGACTATTATTTATCTTTTAATGATTATTAAGATTTTTATTAAAAAAAAAATTCGCTCCTCTTTT
AATGCCTTTATGCAGTTTTTTTTTCCCATTCGATATTTCTATGTTCGGGTTCAGCGTATTTTAAGTTTAATAACTCG
AAAATTCTGCGTTCGTTAAAGCTTTCGAGAAGGATATTATTTCGAAATAAACCGTGTTGTGTAAGCTTGAAGCC
TTTTTGCGCTGCCAATATTCTTATCCATCTATTGTACTCTTTAGATCCAGTATAGTGTATTCTTCCTGCTCCAAGCT
CATCCCACTTGCAACAAAAAAAGTCTAATCTTCTGCAATAATTTCCATCCTTGGCATTCAGAGACATATATTGGT
CAATCGGTTTTAATTTGgtacccaattcgccctatagtgagtcgtattacgcgcgctcactggccgtcgttttacaacgtcgtgactgggaa
aaccctggcgttacccaacttaatcgccttgcagcacatccccctttcgccagctggcgtaatagcgaagaggcccgcaccgatcgcccttccca
acagttgcgcagcctgaatggcgaatggacgcgccctgtagcggcgcattaagcgcggcgggtgtggtggttacgcgcagcgtgaccgctaca
cttgccagcgccctagcgcccgctcctttcgctttcttcccttcctttctcgccacgttcgccggctttccccgtcaagctctaaatcgggggctccct
ttagggttccgatttagtgctttacggcacctcgaccccaaaaaacttgattagggtgatggttcacgtagtgggccatcgccctgatagacggtt
tttcgccctttgacgttggagtccacgttctttaatagtggactcttgttccaaactggaacaacactcaaccctatctcggtctattcttttgatttat
aagggattttgccgatttcggcctattggttaaaaaatgagctgatttaacaaaaatttaacgcgaattttaacaaaatattaacgcttacaatttc
ctgatgcggtattttctccttacgcatctgtgcggtatttcacaccgcatagggtaataactgatataattaaattgaagctctaatttgtgagttta
gtatacatgcatttacttataatacagtttttttagttttgctggccgcatcttctcaaatatgcttcccagcctgcttttctgtaacgttcaccctctacc
ttagcatcccttccctttgcaaatagtcctcttccaacaataataatgtcagatcctgtagagaccacatcatccacggttctatactgttgaccca
atgcgtctcccttgtcatctaaacccacaccgggtgtcataatcaaccaatcgtaaccttcatctcttccacccatgtctctttgagcaataaagcc

gataacaaaatctttgtcgctcttcgcaatgtcaacagtacccttagtatattctccagtagatagggagcccttgcatgacaattctgctaacatc
aaaaggcctctaggttcctttgttacttcttctgccgcctgcttcaaaccgctaacaatacctgggcccaccacaccgtgtgcattcgtaatgtctg
cccattctgctattctgtatacacccgcagagtactgcaatttgactgtattaccaatgtcagcaaattttctgtcttcgaagagtaaaaaattgtac
ttggcggataatgcctttagcggcttaactgtgccctccatggaaaaatcagtcaagatatccacatgtgtttttagtaaacaaattttgggacct
aatgcttcaactaactccagtaattccttggtggtacgaacatccaatgaagcacacaagtttgtttgcttttcgtgcatgatattaaatagcttgg
cagcaacaggactaggatgagtagcagcacgttccttatatgtagctttcgacatgatttatcttcgtttcctgcaggtttttgttctgtgcagttgg
gttaagaatactgggcaatttcatgtttcttcaacactacatatgcgtatatataccaatctaagtctgtgctccttccttcgttcttccttctgttcgg
agattaccgaatcaaaaaaatttcaaggaaaccgaaatcaaaaaaaagaataaaaaaaaaatgatgaattgaaaaggtggtatggtgcact
ctcagtacaatctgctctgatgccgcatagttaagccagccccgacacccgccaacacccgctgacgcgccctgacgggcttgtctgctcccgg
catccgcttacagacaagctgtgaccgtctccgggagctgcatgtgtcagaggttttcaccgtcatcaccgaaacgcgcgag-3’

pMAHS

5’-
acgaaagggcctcgtgatacgcctatttttataggttaatgtcatgataataatggtttcttagtatgatccaatatcaaaggaaatgatagcatt
gaaggatgagactaatccaattgaggagtggcagcatatagaacagctaaagggtagtgctgaaggaagcatacgataccccgcatggaat
gggataatatcacaggaggtactagactacctttcatcctacataaatagacgcatataagtacgcatttaagcataaacacgcactatgccgtt
cttctcatgtatatatatatacaggcaacacgcagatataggtgcgacgtgaacagtgagctgtatgtgcgcagctcgcgttgcattttcggaag
cgctcgttttcggaaacgctttgaagttcctattccgaagttcctattctctagaaagtataggaacttcagagcgcttttgaaaaccaaaagcgct
ctgaagacgcactttcaaaaaaccaaaaacgcaccggactgtaacgagctactaaaatattgcgaataccgcttccacaaacattgctcaaaa
gtatctctttgctatatatctctgtgctatatccctatataacctacccatccacctttcgctccttgaacttgcatctaaactcgacctctacattttt
atgtttatctctagtattactctttagacaaaaaaattgtagtaagaactattcatagagtgaatcgaaaacaatacgaaaatgtaaacatttcct
atacgtagtatatagagacaaaatagaagaaaccgttcataattttctgaccaatgaagaatcatcaacgctatcactttctgttcacaaagtat
gcgcaatccacatcggtatagaatataatcggggatgcctttatcttgaaaaaatgcacccgcagcttcgctagtaatcagtaaacgcgggaag
tggagtcaggctttttttatggaagagaaaatagacaccaaagtagccttcttctaaccttaacggacctacagtgcaaaaagttatcaagaga
ctgcattatagagcgcacaaaggagaaaaaaagtaatctaagatgctttgttagaaaaatagcgctctcgggatgcatttttgtagaacaaaa
aagaagtatagattctttgttggtaaaatagcgctctcgcgttgcatttctgttctgtaaaaatgcagctcagattctttgtttgaaaaattagcgct
ctcgcgttgcatttttgttttacaaaaatgaagcacagattcttcgttggtaaaatagcgctttcgcgttgcatttctgttctgtaaaaatgcagctca
gattctttgtttgaaaaattagcgctctcgcgttgcatttttgttctacaaaatgaagcacagatgcttcgttcaggtggcacttttcggggaaatgt
gcgcggaacccctatttgtttatttttctaaatacattcaaatatgtatccgctcatgagacaataaccctgataaatgcttcaataatattgaaaa
aggaagagtatgagtattcaacatttccgtgtcgcccttattcccttttttgcggcattttgccttcctgtttttgctcacccagaaacgctggtgaaa
gtaaaagatgctgaagatcagttgggtgcacgagtgggttacatcgaactggatctcaacagcggtaagatccttgagagttttcgccccgaag
aacgttttccaatgatgagcacttttaaagttctgctatgtggcgcggtattatcccgtattgacgccgggcaagagcaactcggtcgccgcatac
actattctcagaatgacttggttgagtactcaccagtcacagaaaagcatcttacggatggcatgacagtaagagaattatgcagtgctgccata
accatgagtgataacactgcggccaacttacttctgacaacgatcggaggaccgaaggagctaaccgcttttttgcacaacatgggggatcatg
taactcgccttgatcgttgggaaccggagctgaatgaagccataccaaacgacgagcgtgacaccacgatgcctgtagcaatggcaacaacgt
tgcgcaaactattaactggcgaactacttactctagcttcccggcaacaattaatagactggatggaggcggataaagttgcaggaccacttct
gcgctcggcccttccggctggctggtttattgctgataaatctggagccggtgagcgtgggtctcgcggtatcattgcagcactggggccagatg
gtaagccctcccgtatcgtagttatctacacgacggggagtcaggcaactatggatgaacgaaatagacagatcgctgagataggtgcctcact
gattaagcattggtaactgtcagaccaagtttactcatatatactttagattgatttaaaacttcatttttaatttaaaaggatctaggtgaagatcc
tttttgataatctcatgaccaaaatcccttaacgtgagttttcgttccactgagcgtcagaccccgtagaaaagatcaaaggatcttcttgagatcc
tttttttctgcgcgtaatctgctgcttgcaaacaaaaaaaccaccgctaccagcggtggtttgtttgccggatcaagagctaccaactctttttccg
aaggtaactggcttcagcagagcgcagataccaaatactgttcttctagtgtagccgtagttaggccaccacttcaagaactctgtagcaccgcc
tacatacctcgctctgctaatcctgttaccagtggctgctgccagtggcgataagtcgtgtcttaccgggttggactcaagacgatagttaccgga
taaggcgcagcggtcgggctgaacggggggttcgtgcacacagcccagcttggagcgaacgacctacaccgaactgagatacctacagcgtg
agctatgagaaagcgccacgcttcccgaagggagaaaggcggacaggtatccggtaagcggcagggtcggaacaggagagcgcacgagg
gagcttccagggggaaacgcctggtatctttatagtcctgtcgggtttcgccacctctgacttgagcgtcgatttttgtgatgctcgtcaggggggc

ggagcctatggaaaaacgccagcaacgcggcctttttacggttcctggccttttgctggccttttgctcacatgttctttcctgcgttatcccctgatt
ctgtggataaccgtattaccgcctttgagtgagctgataccgctcgccgcagccgaacgaccgagcgcagcgagtcagtgagcgaggaagcgg
aagagcgcccaatacgcaaaccgcctctccccgcgcgttggccgattcattaatgcagctggcacgacaggtttcccgactggaaagcgggca
gtgagcgcaacgcaattaatgtgagttagctcactcattaggcaccccaggctttacactttatgcttccggctcgtatgttgtgtggaattgtgag
cggataacaatttcacacaggaaacagctatgaccatgattacgccaagcgcgcaattaaccctcactaaagggaacaaaagctggAGCTC
Agccacaatagaagctttctaactgatctatccaaaactgaaaattacattcttgattaggtttatcacaggcaaatgtaatttgtggtattttgcc
gttcaaaatctgtagaattttctcattggtcacattacaacctgaaaatactttatctacaatcataccattcttataacatgtccccttaatactag
gatcaggcatgaacgcatcacagacaaaatcttcttgacaaacgtcacaattgatccctccccatccgttatcacaatgacaggtgtcattttgtg
ctcttatgggacgatccttattaccgctttcatccggtgatagaccgccacagaggggcagagagcaatcatcacctgcaaacccttctatacac
tcacatctaccagtgtacgaattgcattcagaaaactgtttgcattcaaaaataggtagcatacaattaaaacatggcgggcatgtatcattgcc
cttatcttgtgcagttagacgcgaattttcgaagaagtaccttcaaagaatggggtcttatcttgttttgcaagtaccactgagcaggataataat
agaaatgataatatactatagtagagataacgtcgatgacttcccatactgtaattgcttttagttgtgtatttttagtgtgcaagtttctgtaaatc
gattaatttttttttctttcctctttttattaaccttaatttttattttagattcctgacttcaactcaagacgcacagatattataacatctgcataatag
gcatttgcaagaattactcgtgagtaaggaaagagtgaggaactatcgcatacctgcatttaaagatgccgatttgggcgcgaatcctttattttg
gcttcaccctcatactattatcagggccagaaaaaggaagtgtttccctccttcttgaattgatgttaccctcataaagcacgtggcctcttatcga
gaaagaaattaccgtcgctcgtgatttgtttgcaaaaagaacaaaactgaaaaaacccagacacgctcgacttcctgtcttcctattgattgcag
cttccaatttcgtcacacaacaaggtcctagcgacggctcacaggttttgtaacaagcaatcgaaggttctggaatggcgggaaagggtttagt
accacatgctatgatgcccactgtgatctccagagcaaagttcgttcgatcgtactgttactctctctctttcaaacagaattgtccgaatcgtgtg
acaacaacagcctgttctcacacactcttttcttctaaccaagggggtggtttagtttagtagaacctcgtgaaacttacatttacatatatataaa
cttgcataaattggtcaatgcaagaaatacatatttggtcttttctaattcgtagtttttcaagttcttagatgctttctttttctctttttttacagatcaT
CAAGGAAGTAATTATCTACTTTTTACAACAAATATAAAACAatgtccagatacctgctgcgcgatgtccaggctgtattacgcg
gagttcgcaaagtggccgagagtagcttaaagctggagacggagaaagtcagtctgcggcttggtgactttcggtcacagccttcccttcgcag
tgtgcctgcttccctcacaagtcgatcacaggcatttagcctacaggagatagctgctcgtgccggagttgttctgcgaggagtgcaacaacagt
tccgaaacgtcactggagtgaatgccgctcctgttgtagcctttgataatggatcagttctatacagtgaaagaatccactcgcagagttcgcag
aagcaggccccgactacagtaccaacaggatccgtcagcaattcccctcaaccggaaggaaaggcaaacgaagctgctgaacgcgcaaaac
agtttatgaatcctccagttgcgccaatggatcctgtcgacaagaatgaatttgtcgccatgccggagatgggtcgtagtaatggaaatggaga
aaacaaacaagctgctgatttcatgaaaaaccaaggtgacaccgatatggattcccagtacgcgcctgattcatcgaagaacacgaaatcggt
tcccacgaaggaaatcgttgctgaagatggttcgatgagcattgaggatatcaagaaagctacgcaggttactcctggagttgcagttaaaaac
gagggtgtttaaATATTGAATTGAATTGAAATCGATAGATCAATTTTTTTCTTTTCTCTTTCCCCATCCTTTACGCTAA
AATAATAGTTTATTTTATTTTTTGAATATTTTTTATTTATATACGTATATATAGACTATTATTTATCTTTTAATGATTATT
AAGATTTTTATTAAAAAAAAATTCGCTCCTCTTTTAATGCCTTTATGCAGTTTTTTTTTCCCATTCGATATTTCTAT
GTTCGGGTTCAGCGTATTTTAAGTTTAATAACTCGAAAATTCTGCGTTCGTTAAAGCTTTCGAGAAGGATATTAT
TTCGAAATAAACCGTGTTGTGTAAGCTTGAAGCCTTTTTGCGCTGCCAATATTCTTATCCATCTATTGTACTCTTT
AGATCCAGTATAGTGTATTCTTCCTGCTCCAAGCTCATCCCACTTGCAACAAAAAAAGTCTAATCTTCTGCAATA
ATTTCCATCCTTGGCATTCAGAGACATATATTGGTCAATCGGTTTTAATTTGgtacccaattcgccctatagtgagtcgtatt
acgcgcgctcactggccgtcgttttacaacgtcgtgactgggaaaaccctggcgttacccaacttaatcgccttgcagcacatccccctttcgcca
gctggcgtaatagcgaagaggcccgcaccgatcgcccttcccaacagttgcgcagcctgaatggcgaatggacgcgccctgtagcggcgcatt
aagcgcggcgggtgtggtggttacgcgcagcgtgaccgctacacttgccagcgccctagcgcccgctcctttcgctttcttcccttcctttctcgcc
acgttcgccggctttccccgtcaagctctaaatcgggggctccctttagggttccgatttagtgctttacggcacctcgaccccaaaaaacttgatt
agggtgatggttcacgtagtgggccatcgccctgatagacggtttttcgccctttgacgttggagtccacgttctttaatagtggactcttgttccaa
actggaacaacactcaaccctatctcggtctattcttttgatttataaggtattttgccgatttcggcctattggttaaaaaatgagctgatttaaca
aaaatttaacgcgaattttaacaaaatattaacgcttacaatttcctgatgcggtattttctccttacgcatctgtgcggtatttcacaccgcatagg
gtaataactgatataattaaattgaagctctaatttgtgagtttagtatacatgcatttacttataatacagtttttttagttttgctggccgcatcttct
caaatatgcttcccagcctgcttttctgtaacgttcaccctctaccttagcatcccttccctttgcaaatagtcctcttccaacaataataatgtcaga
tcctgtagagaccacatcatccacggttctatactgttgacccaatgcgtctcccttgtcatctaaacccacaccgggtgtcataatcaaccaatc
gtaaccttcatctcttccacccatgtctctttgagcaataaagccgataacaaaatctttgtcgctcttcgcaatgtcaacagtacccttagtatatt
ctccagtagatagggagcccttgcatgacaattctgctaacatcaaaaggcctctaggttcctttgttacttcttctgccgcctgcttcaaaccgct
aacaatacctgggcccaccacaccgtgtgcattcgtaatgtctgcccattctgctattctgtatacacccgcagagtactgcaatttgactgtatta

ccaatgtcagcaaattttctgtcttcgaagagtaaaaaattgtacttggcggataatgcctttagcggcttaactgtgccctccatggaaaaatca
gtcaagatatccacatgtgtttttagtaaacaaattttgggacctaatgcttcaactaactccagtaattccttggtggtacgaacatccaatgaa
gcacacaagtttgtttgcttttcgtgcatgatattaaatagcttggcagcaacaggactaggatgagtagcagcacgttccttatatgtagctttcg
acatgatttatcttcgtttcctgcaggtttttgttctgtgcagttgggttaagaatactgggcaatttcatgtttcttcaacactacatatgcgtatata
taccaatctaagtctgtgctccttccttcgttcttccttctgttcggagattaccgaatcaaaaaaatttcaaggaaaccgaaatcaaaaaaaaga
ataaaaaaaaaatgatgaattgaaaaggtggtatggtgcactctcagtacaatctgctctgatgccgcatagttaagccagccccgacacccg
ccaacacccgctgacgcgccctgacgggcttgtctgctcccggcatccgcttacagacaagctgtgaccgtctccgggagctgcatgtgtcaga
ggttttcaccgtcatcaccgaaacgcgcgag-3’

pRvLEAM

5’-
acgaaagggcctcgtgatacgcctatttttataggttaatgtcatgataataatggtttcttagtatgatccaatatcaaaggaaatgatagcatt
gaaggatgagactaatccaattgaggagtggcagcatatagaacagctaaagggtagtgctgaaggaagcatacgataccccgcatggaat
gggataatatcacaggaggtactagactacctttcatcctacataaatagacgcatataagtacgcatttaagcataaacacgcactatgccgtt
cttctcatgtatatatatatacaggcaacacgcagatataggtgcgacgtgaacagtgagctgtatgtgcgcagctcgcgttgcattttcggaag
cgctcgttttcggaaacgctttgaagttcctattccgaagttcctattctctagaaagtataggaacttcagagcgcttttgaaaaccaaaagcgct
ctgaagacgcactttcaaaaaaccaaaaacgcaccggactgtaacgagctactaaaatattgcgaataccgcttccacaaacattgctcaaaa
gtatctctttgctatatatctctgtgctatatccctatataacctacccatccacctttcgctccttgaacttgcatctaaactcgacctctacatttttt
atgtttatctctagtattactctttagacaaaaaaattgtagtaagaactattcatagagtgaatcgaaaacaatacgaaaatgtaaacatttcct
atacgtagtatatagagacaaaatagaagaaaccgttcataattttctgaccaatgaagaatcatcaacgctatcactttctgttcacaaagtat
gcgcaatccacatcggtatagaatataatcggggatgcctttatcttgaaaaaatgcacccgcagcttcgctagtaatcagtaaacgcgggaag
tggagtcaggctttttttatggaagagaaaatagacaccaaagtagccttcttctaaccttaacggacctacagtgcaaaaagttatcaagaga
ctgcattatagagcgcacaaaggagaaaaaaagtaatctaagatgctttgttagaaaaatagcgctctcgggatgcatttttgtagaacaaaa
aagaagtatagattctttgttggtaaaatagcgctctcgcgttgcatttctgttctgtaaaaatgcagctcagattctttgtttgaaaaattagcgct
ctcgcgttgcatttttgttttacaaaaatgaagcacagattcttcgttggtaaaatagcgctttcgcgttgcatttctgttctgtaaaaatgcagctca
gattctttgtttgaaaaattagcgctctcgcgttgcatttttgttctacaaaatgaagcacagatgcttcgttcaggtggcacttttcggggaaatgt
gcgcggaacccctatttgtttatttttctaaatacattcaaatatgtatccgctcatgagacaataaccctgataaatgcttcaataatattgaaaa
aggaagagtatgagtattcaacatttccgtgtcgcccttattcccttttttgcggcattttgccttcctgtttttgctcacccagaaacgctggtgaaa
gtaaaagatgctgaagatcagttgggtgcacgagtgggttacatcgaactggatctcaacagcggtaagatccttgagagttttcgccccgaag
aacgttttccaatgatgagcacttttaaagttctgctatgtggcgcggtattatcccgtattgacgccgggcaagagcaactcggtcgccgcatac
actattctcagaatgacttggttgagtactcaccagtcacagaaaagcatcttacggatggcatgacagtaagagaattatgcagtgctgccata
accatgagtgataacactgcggccaacttacttctgacaacgatcggaggaccgaaggagctaaccgcttttttgcacaacatgggggatcatg
taactcgccttgatcgttgggaaccggagctgaatgaagccataccaaacgacgagcgtgacaccacgatgcctgtagcaatggcaacaacgt
tgcgcaaactattaactggcgaactacttactctagcttcccggcaacaattaatagactggatggaggcggataaagttgcaggaccacttct
gcgctcggcccttccggctggctggtttattgctgataaatctggagccggtgagcgtgggtctcgcggtatcattgcagcactggggccagatg
gtaagccctcccgtatcgtagttatctacacgacggggagtcaggcaactatggatgaacgaaatagacagatcgctgagataggtgcctcact
gattaagcattggtaactgtcagaccaagtttactcatatatactttagattgatttaaaacttcatttttaatttaaaaggatctaggtgaagatcc
tttttgataatctcatgaccaaaatcccttaacgtgagttttcgttccactgagcgtcagaccccgtagaaaagatcaaaggatcttcttgagatcc
tttttttctgcgcgtaatctgctgcttgcaaacaaaaaaaccaccgctaccagcggtggtttgtttgccggatcaagagctaccaactctttttccg
aaggtaactggcttcagcagagcgcagataccaaatactgttcttctagtgtagccgtagttaggccaccacttcaagaactctgtagcaccgcc
tacatacctcgctctgctaatcctgttaccagtggctgctgccagtggcgataagtcgtgtcttaccgggttggactcaagacgatagttaccgga
taaggcgcagcggtcgggctgaacggggggttcgtgcacacagcccagcttggagcgaacgacctacaccgaactgagatacctacagcgtg
agctatgagaaagcgccacgcttcccgaagggagaaaggcggacaggtatccggtaagcggcagggtcggaacaggagagcgcacgagg
gagcttccagggggaaacgcctggtatctttatagtcctgtcgggtttcgccacctctgacttgagcgtcgatttttgtgatgctcgtcaggggggc
ggagcctatggaaaaacgccagcaacgcggcctttttacggttcctggccttttgctggccttttgctcacatgttctttcctgcgttatcccctgatt
ctgtggataaccgtattaccgcctttgagtgagctgataccgctcgccgcagccgaacgaccgagcgcagcgagtcagtgagcgaggaagcgg

aagagcgcccaatacgcaaaccgcctctccccgcgcgttggccgattcattaatgcagctggcacgacaggtttcccgactggaaagcgggca
gtgagcgcaacgcaattaatgtgagttagctcactcattaggcaccccaggctttacactttatgcttccggctcgtatgttgtgtggaattgtgag
cggataacaatttcacacaggaaacagctatgaccatgattacgccaagcgcgcaattaaccctcactaaagggaacaaaagctggAGCTC
Agccacaatagaagctttctaactgatctatccaaaactgaaaattacattcttgattaggtttatcacaggcaaatgtaatttgtggtattttgcc
gttcaaaatctgtagaattttctcattggtcacattacaacctgaaaatactttatctacaatcataccattcttataacatgtccccttaatactag
gatcaggcatgaacgcatcacagacaaaatcttcttgacaaacgtcacaattgatccctccccatccgttatcacaatgacaggtgtcattttgtg
ctcttatgggacgatccttattaccgctttcatccggtgatagaccgccacagaggggcagagagcaatcatcacctgcaaacccttctatacac
tcacatctaccagtgtacgaattgcattcagaaaactgtttgcattcaaaaataggtagcatacaattaaaacatggcgggcatgtatcattgcc
cttatcttgtgcagttagacgcgaattttcgaagaagtaccttcaaagaatggggtcttatcttgttttgcaagtaccactgagcaggataataat
agaaatgataatatactatagtagagataacgtcgatgacttcccatactgtaattgcttttagttgtgtatttttagtgtgcaagtttctgtaaatc
gattaattttttttctttcctcttttattaaccttaattttattttagattcctgacttcaactcaagacgcacagatattataacatctgcataatag
gcatttgcaagaattactcgtgagtaaggaaagagtgaggaactatcgcatacctgcatttaaagatgccgatttgggcgcgaatcctttattttg
gcttcaccctcatactattatcagggccagaaaaaggaagtgtttccctccttcttgaattgatgttaccctcataaagcacgtggcctcttatcga
gaaagaaattaccgtcgctcgtgatttgtttgcaaaaagaacaaaactgaaaaaacccagacacgctcgacttcctgtcttcctattgattgcag
cttccaatttcgtcacacaacaaggtcctagcgacggctcacaggttttgtaacaagcaatcgaaggttctggaatggcgggaaagggtttagt
accacatgctatgatgcccactgtgatctccagagcaaagttcgttcgatcgtactgttactctctctctttcaaacagaattgtccgaatcgtgtg
acaacaacagcctgttctcacacactcttttcttctaaccaagggggtggtttagtttagtagaacctcgtgaaacttacatttacatatatataaa
cttgcataaattggtcaatgcaagaaatacatatttggtcttttctaattcgtagtttttcaagttcttagatgctttctttttctctttttttacagatcaT
CAAGGAAGTAATTATCTACTTTTTACAACAAATATAAAACAatgtttctcgcccgaaacgctggacgcgctggatatcgaggtg
ttgtagcttaccagcaagcagcctctttctctgtttcatcagctaaggctgctggatcacggtcaagcggtggctccgacgctggcgattatgcta
gggaggccgcggaacatgccaaagctggattgaaggatctcaagaatgaagcctcgtggaaagccaaaggtgtcgctaaccaagccgctgg
agcattcgaacgagcgaaggatacagtgaaggaaggtgtccatgacatgaagcgcagtggaagccgcgtcttcgaacaaggacaggaggaa
gtagaggctggtgctcaacacgcaaaggctggctatcagagtgccaagaatgctgcgcaagacactgccgcaactcttaaagacaaagccgg
cagcgcctggaatcaagcgaaacacgttgtggaagataagggagaggatgttgttgaagcagtcaaggacactgcttcaaaagtctggggca
aagccaaacatgtggccgaggacgttaaggagaacgcgcaatcaccaggtggcattgcggataaggcctccgatgtgtggtcagcggccaag
gataaagctgctgatgtcttgtcgggagctaagcacactgctgagaacctagctcacaaagcccaagccgccatccacgatgctacagcttctt
caggctcgcagtcgcagtcgcagtcccagtctcagtatcgacaaggacaacaacagggaagacaagatcagcagcaaagcaagagccagtg
gggacagactagcccgcagtctccggatggattccgccctcaagccggccagggaccacaaggaggcaagggacctggtcaagctggcggt
cgacgataaATATTGAATTGAATTGAAATCGATAGATCAATTTTTTTCTTTTCTCTTTCCCCATCCTTTACGCTAAAA
TAATAGTTTATTTTATTTTTTGAATATTTTTTATTTATATACGTATATATAGACTATTATTTATCTTTTAATGATTATTAA
GATTTTTATTAAAAAAAAATTCGCTCCTCTTTTAATGCCTTTATGCAGTTTTTTTTTCCCATTCGATATTTCTATGT
TCGGGTTCAGCGTATTTTAAGTTTAATAACTCGAAAATTCTGCGTTCGTTAAAGCTTTCGAGAAGGATATTATTT
CGAAATAAACCGTGTTGTGTAAGCTTGAAGCCTTTTTGCGCTGCCAATATTCTTATCCATCTATTGTACTCTTTAG
ATCCAGTATAGTGTATTCTTCCTGCTCCAAGCTCATCCCACTTGCAACAAAAAAAGTCTAATCTTCTGCAATAATT
TCCATCCTTGGCATTCAGAGACATATATTGGTCAATCGGTTTTAATTTGgtacccaattcgccctatagtgagtcgtattacg
cgcgctcactggccgtcgttttacaacgtcgtgactgggaaaaccctggcgttacccaacttaatcgccttgcagcacatccccctttcgccagct
ggcgtaatagcgaagaggcccgcaccgatcgcccttcccaacagttgcgcagcctgaatggcgaatggacgcgccctgtagcggcgcattaa
gcgcggcgggtgtggtggttacgcgcagcgtgaccgctacacttgccagcgccctagcgcccgctcctttcgctttcttcccttcctttctcgccac
gttcgccggctttccccgtcaagctctaaatcgggggctccctttagggttccgatttagtgctttacggcacctcgaccccaaaaaacttgattag
ggtgatggttcacgtagtgggccatcgccctgatagacggtttttcgccctttgacgttggagtccacgttctttaatagtggactcttgttccaaac
tggaacaacactcaaccctatctcggtctattcttttgatttataagggattttgccgatttcggcctattggttaaaaaatgagctgatttaacaaa
aatttaacgcgaattttaacaaaatattaacgcttacaatttcctgatgcggtattttctccttacgcatctgtgcggtatttcacaccgcatagggt
aataactgatataattaaattgaagctctaatttgtgagtttagtatacatgcatttacttataatacagtttttttagttttgctggccgcatcttctca
aatatgcttcccagcctgcttttctgtaacgttcaccctctaccttagcatcccttccctttgcaaatagtcctcttccaacaataataatgtcagatc
ctgtagagaccacatcatccacggttctatactgttgacccaatgcgtctcccttgtcatctaaacccacaccgggtgtcataatcaaccaatcgt
aaccttcatctcttccacccatgtctctttgagcaataaagccgataacaaaatctttgtcgctcttcgcaatgtcaacagtacccttagtatattct
ccagtagatagggagcccttgcatgacaattctgctaacatcaaaaggcctctaggttcctttgttacttcttctgccgcctgcttcaaaccgctaa
caatacctgggcccaccacaccgtgtgcattcgtaatgtctgcccattctgctattctgtatacacccgcagagtactgcaatttgactgtattacc

aatgtcagcaaattttctgtcttcgaagagtaaaaaattgtacttggcggataatgcctttagcggcttaactgtgccctccatggaaaaatcagt
caagatatccacatgtgtttttagtaaacaaattttgggacctaatgcttcaactaactccagtaattccttggtggtacgaacatccaatgaagc
acacaagtttgtttgcttttcgtgcatgatattaaatagcttggcagcaacaggactaggatgagtagcagcacgttccttatatgtagctttcgac
atgatttatcttcgtttcctgcaggtttttgttctgtgcagttgggttaagaatactgggcaatttcatgtttcttcaacactacatatgcgtatatata
ccaatctaagtctgtgctccttccttcgttcttccttctgttcggagattaccgaatcaaaaaaatttcaaggaaaccgaaatcaaaaaaaagaat
aaaaaaaaaatgatgaattgaaaaggtggtatggtgcactctcagtacaatctgctctgatgccgcatagttaagccagccccgacacccgcc
aacacccgctgacgcgccctgacgggcttgtctgctcccggcatccgcttacagacaagctgtgaccgtctccgggagctgcatgtgtcagagg
ttttcaccgtcatcaccgaaacgcgcgag-3’

pCAHS3-GFP

5’-
acgaaagggcctcgtgatacgcctatttttataggttaatgtcatgataataatggtttcttagtatgatccaatatcaaaggaaatgatagcatt
gaaggatgagactaatccaattgaggagtggcagcatatagaacagctaaagggtagtgctgaaggaagcatacgataccccgcatggaat
gggataatatcacaggaggtactagactacctttcatcctacataaatagacgcatataagtacgcatttaagcataaacacgcactatgccgtt
cttctcatgtatatatatatacaggcaacacgcagatataggtgcgacgtgaacagtgagctgtatgtgcgcagctcgcgttgcattttcggaag
cgctcgttttcggaaacgctttgaagttcctattccgaagttcctattctctagaaagtataggaacttcagagcgcttttgaaaaccaaaagcgct
ctgaagacgcactttcaaaaaaccaaaaacgcaccggactgtaacgagctactaaaatattgcgaataccgcttccacaaacattgctcaaaa
gtatctctttgctatatatctctgtgctatatccctatataacctacccatccacctttcgctccttgaacttgcatctaaactcgacctctacattttt
atgtttatctctagtattactctttagacaaaaaaattgtagtaagaactattcatagagtgaatcgaaaacaatacgaaaatgtaaacatttcct
atacgtagtatatagagacaaaatagaagaaaccgttcataatttctgaccaatgaagaatcatcaacgctatcactttctgttcacaaagtat
gcgcaatccacatcggtatagaatataatcggggatgcctttatcttgaaaaaatgcacccgcagcttcgctagtaatcagtaaacgcgggaag
tggagtcaggctttttttatggaagagaaaatagacaccaaagtagccttcttctaaccttaacggacctacagtgcaaaaagttatcaagaga
ctgcattatagagcgcacaaaggagaaaaaaagtaatctaagatgctttgttagaaaaatagcgctctcgggatgcatttttgtagaacaaaa
aagaagtatagattctttgttggtaaaatagcgctctcgcgttgcatttctgttctgtaaaaatgcagctcagattctttgtttgaaaaattagcgct
ctcgcgttgcatttttgttttacaaaaatgaagcacagattcttcgttggtaaaatagcgctttcgcgttgcatttctgttctgtaaaaatgcagctca
gattctttgtttgaaaaattagcgctctcgcgttgcatttttgttctacaaaatgaagcacagatgcttcgttcaggtggcacttttcggggaaatgt
gcgcggaacccctatttgtttatttttctaaatacattcaaatatgtatccgctcatgagacaataaccctgataaatgcttcaataatattgaaaa
aggaagagtatgagtattcaacatttccgtgtcgcccttattcccttttttgcggcattttgccttcctgtttttgctcacccagaaacgctggtgaaa
gtaaaagatgctgaagatcagttgggtgcacgagtgggttacatcgaactggatctcaacagcggtaagatccttgagagttttcgccccgaag
aacgttttccaatgatgagcacttttaaagttctgctatgtggcgcggtattatcccgtattgacgccgggcaagagcaactcggtagccgcatac
actattctcagaatgacttggttgagtactcaccagtcacagaaaagcatcttacggatggcatgacagtaagagaattatgcagtgctgccata
accatgagtgataacactgcggccaacttacttctgacaacgatcggaggaccgaaggagctaaccgcttttttgcacaacatgggggatcatg
taactcgccttgatcgttgggaaccggagctgaatgaagccataccaaacgacgagcgtgacaccacgatgcctgtagcaatggcaacaacgt
tgcgcaaactattaactggcgaactacttactctagcttcccggcaacaattaatagactggatggaggcggataaagttgcaggaccacttct
gcgctcggcccttccggctggctggtttattgctgataaatctggagccggtgagcgtgggtctcgcggtatcattgcagcactggggccagatg
gtaagccctcccgtatcgtagttatctacacgacggggagtcaggcaactatggatgaacgaaatagacagatcgctgagataggtgcctcact
gattaagcattggtaactgtcagaccaagtttactcatatatactttagattgatttaaaacttcatttttaatttaaaaggatctaggtgaagatcc
tttttgataatctcatgaccaaaatcccttaacgtgagttttcgttccactgagcgtcagaccccgtagaaaagatcaaaggatcttcttgagatcc
tttttttctgcgcgtaatctgctgcttgcaaacaaaaaaaccaccgctaccagcggtggtttgtttgccggatcaagagctaccaactctttttccg
aaggtaactggcttcagcagagcgcagataccaaatactgttcttctagtgtagccgtagttaggccaccacttcaagaactctgtagcaccgcc
tacatacctcgctctgctaatcctgttaccagtggctgctgccagtggcgataagtcgtgtcttaccgggttggactcaagacgatagttaccgga
taaggcgcagcggtcgggctgaacggggggttcgtgcacacagcccagcttggagcgaacgacctacaccgaactgagatacctacagcgtg
agctatgagaaagcgccacgcttcccgaagggagaaaggcggacaggtatccggtaagcggcagggtcggaacaggagagcgcacgagg
gagcttccagggggaaacgcctggtatctttatagtcctgtcgggtttcgccacctctgacttgagcgtcgatttttgtgatgctcgtcaggggggc
ggagcctatggaaaaacgccagcaacgcggcctttttacggttcctggccttttgctggccttttgctcacatgttctttcctgcgttatcccctgatt
ctgtggataaccgtattaccgcctttgagtgagctgataccgctcgccgcagccgaacgaccgagcgcagcgagtcagtgagcgaggaagcgg

aagagcgcccaatacgcaaaccgcctctccccgcgcgttggccgattcattaatgcagctggcacgacaggtttcccgactggaaagcgggca
gtgagcgcaacgcaattaatgtgagttagctcactcattaggcaccccaggctttacactttatgcttccggctcgtatgttgtgtggaattgtgag
cggataacaatttcacacaggaaacagctatgaccatgattacgccaagcgcgcaattaaccctcactaaagggaacaaaagctggAGCTC
Agccacaatagaagctttctaactgatctatccaaaactgaaaattacattcttgattaggtttatcacaggcaaatgtaatttgtggtattttgcc
gttcaaaatctgtagaattttctcattggtaacattacaacctgaaaatactttatctacaatcataccattcttataacatgtccccttaatactag
gatcaggcatgaacgcatcgcagacaaaatcttcttgacaaacgtcacaattgatccctccccatccgttatcacaatgacaggtgtcattttgtg
ctcttatgggacgatccttattaccgctttcatccggtgatagaccgccacagaggggcagagagcaatcatcacctgcaaacccttctatacac
tcacatctaccagtgtacgaattgcattcagaaaactgtttgcattcaaaaataggtagcatacaattaaaacatggcgggcatgtatcattgcc
cttatcttgtgcagttagacgcgaattttcgaagaagtaccttcaaagaatggggtcttatcttgttttgcaagtaccactgagcaggataataat
agaaatgataatatactatagtagagataacgtcgatgacttcccatactgtaattgcttttagttgtgtatttttagtgtgcaagtttctgtaaatc
gattaatttttttctttcctcttttattaaccttaattttattttagattcctgacttcaactcaagacgcacagatattataacatctgcataatag
gcatttgcaagaattactcgtgagtaaggaaagagtgaggaactatcgcatacctgcatttaaagatgccgatttgggcgcgaatcctttattttg
gcttcaccctcatactattatcagggccagaaaaaggaagtgtttccctccttcttgaattgatgttaccctcataaagcacgtggcctcttatcga
gaaagaaattaccgtcgctcgtgatttgtttgcaaaaagaacaaaactgaaaaaacccagacacgctcgacttcctgtcttcctattgattgcag
cttccaatttcgtcacacaacaaggtcctagcgacggctcacaggttttgtaacaagcaatcgaaggttctggaatggcgggaaagggtttagt
accacatgctatgatgcccactgtgatctccagagcaaagttcgttcgatcgtactgttactctctctctttcaaacagaattgtccgaatcgtgtg
acaacaacagcctgttctcacacactcttttcttctaaccaagggggtggtttagtttagtagaacctcgtgaaacttacatttacatatatataaa
cttgcataaattggtcaatgcaagaaatacatatttggtcttttctaattcgtagtttttcaagttcttagatgctttctttttctctttttacagatcaT
CAAGGAAGTAATTATCTACTTTTTACAACAAATATAAAACAatgtcttcccgacagaaccagcaatcgtccagccaacactcgt
cctccagccagcaaggtggtcaaggtggtcaaggtgttcaaggaagttccagctactcgcgcaccgaggtccacaccagcagtggaggacct
accatcggtggagcccagcgaactgtccccgtccccccctggatctcactccgaggtccatgaggagcgtgaggtcatcaagcatggtaccaaa
accgaaagcgagacccacgtcgtcaccgtcccagtgacaactttcggcagcaccaacatggaatctgtccgaaccggcttcaccgtcacccaa
gacaagaacttgaccgttgctgctcccaacatcgctgctcccatccacagcaacctcgaccttaacctcggcggtggagctcgcgctgaaatta
ccgcagggaccaccgttgacttgagcaagatccagcgcaaggatttgggacctgaagagtatgctcgctacaaggccaaggtcgagcaactg
gccaggcaagatgagcaagacgcgggtatgcgcgctgcccagtaccgagaggaagtggagcgtgatgccgaactcatccgacagatcttgga
gcgacaacacatccgtgatcttgaattccgcaaggaaatggttgagaaccaagtcaaccgacaagagagagaaatccagctggaggctgagt
acgcaatgcgagccctcgagcttgagcgcaatgccgctaaagaggctttggaaagcgccaaggcccagactaacgtcaatgtcaaggtcgag
tccgctattggtaccaccgtctccaagggtgcaatccagacttccgccgacaagagcagcaccaccaagactggacccaccaccgtcactcag
attaaacataccgaacaacacactgaacgccgagatccaccggtcatggtgagcaagggcgccgagctgttcaccggcatcgtgcccatcctg
atcgagctgaatggcgatgtgaatggccacaagttcagcgtgagcggcgagggcgagggcgatgccacctacggcaagctgaccctgaagtt
catctgcaccaccggcaagctgcctgtgccctggcccaccctggtgaccaccctgagctacggcgtgcagtgcttctcacgctaccccgatcac
atgaagcagcacgacttcttcaagagcgccatgcctgagggctacatccaggagcgcaccatcttcttcgaggatgacggcaactacaagtcg
cgcgccgaggtgaagttcgagggcgataccctggtgaatcgcatcgagctgaccggcaccgatttcaaggaggatggcaacatcctgggcaa
taagatggagtacaactacaacgcccacaatgtgtacatcatgaccgacaaggccaagaatggcatcaaggtgaacttcaagatccgccaca
acatcgaggatggcagcgtgcagctggccgaccactaccagcagaatacccccatcggcgatggccctgtgctgctgcccgataaccactacc
tgtccacccagagcgccctgtccaaggaccccaacgagaagcgcgatcacatgatctacttcggcttcgtgaccgccgccgccatcacccacg
gcatggatgagctgtacaagtaaATATTGAATTGAATTGAAATCGATAGATCAATTTTTTTCTTTTCTCTTTCCCCATCaT
TTACGCTAAAATAATAGTTTATTTTATTTTTTGAATATTTTTTATTTATATACGTATATATAGACTATTATTTATCTTTT
AATGATTATTAAGATTTTTATTAAAAAAAAATTCGCTCCTCTTTTAATGCCTTTATGCAGTTTTTTTTTCCCATTCG
ATATTTCTATGTTCGGGTTCAGCGTATTTTAAGTTTAATAACTCGAAAATTCTGCGTTCGTTAAAGCTTTCGAGA
AGGATATTATTTCGAAATAAACCGTGTTGTGTAAGCTTGAAGCCTTTTTGCGCTGCCAATATTCTTATCCATCTAT
TGTACTCTTTAGATCCAGTATAGTGTATTCTTCCTGCTCCAAGCTCATCCCACTTGCAACAAAAAAAGTCTAATCT
TCTGCAATAATTTCCATCCTTGGCATTCAGAGACATATATTGGTCAATCGGTTTTAATTTGgtacccaattcgccctata
gtgagtcgtattacgcgcgctcactggccgtcgttttacaacgtcgtgactgggaaaaccctggcgttacccaacttaatcgccttgcagcacatc
cccctttcgccagctggcgtaatagcgaagaggcccgcaccgatcgcccttcccaacagttgcgcagcctgaatggcgaatggacgcgccctgt
agcggcgcattaagcgcggcgggtgtggtggttacgcgcagcgtgaccgctacacttgccagcgccctagcgcccgctcctttcgctttcttccct
tcctttctcgccacgttcgccggctttccccgtcaagctctaaatcgggggctccctttagggttccgatttagtgctttacggcacctcgaccccaa
aaaacttgattagggtgatggttcacgtagtgggccatcgccctgatagacggtttttcgccctttgacgttggagtccacgttctttaatagtgga

ctcttgttccaaactggaacaacactcaaccctatctcggtctattcttttgatttataagggattttgccgatttcggcctattggttaaaaaatga
gctgatttaacaaaaatttaacgcgaattttaacaaaatattaacgcttacaatttcctgatgcggtattttctccttacgcatctgtgcggtatttca
caccgcatagggtaataactgatataattaaattgaagctctaatttgtgagtttagtatacatgcatttacttataatacagtttttttagttttgctg
gccgcatcttctcaaatatgcttcccagcctgcttttctgtaacgttcaccctctaccttagcatcccttccctttgcaaatagtcctcttccaacaat
aataatgtcagatcctgtagagaccacatcatccacggttctatactgttgacccaatgcgtctcccttgtcatctaaacccacaccgggtgtcat
aatcaaccaatcgtaaccttcatctcttccacccatgtctctttgagcaataaagccgataacaaaatctttgtcgctcttcgcaatgtcaacagta
cccttagtatattctccagtagatagggagcccttgcatgacaattctgctaacatcaaaaggcctctaggttcctttgttacttcttctgccgcctg
cttcaaaccgctaacaatacctgggcccaccacaccgtgtgcattcgtaatgtctgcccattctgctattctgtatacacccgcagagtactgcaa
tttgactgtattaccaatgtcagcaaattttctgtcttcgaagagtaaaaaattgtacttggcggataatgcctttagcggcttaactgtgccctcca
tggaaaaatcagtcaagatatccacatgtgtttttagtaaacaaattttgggacctaatgcttcaactaactccagtaattccttggtggtacgaa
catccaatgaagcacacaagtttgtttgcttttcgtgcatgatattaaatagcttggcagcaacaggactaggatgagtagcagcacgttccttat
atgtagctttcgacatgatttatcttcgtttcctgcaggtttttgttctgtgcagttgggttaagaatactgggcaatttcatgtttcttcaacactaca
tatgcgtatatataccaatctaagtctgtgctccttccttcgttcttccttctgttcggagattaccgaatcaaaaaaatttcaaggaaaccgaaatc
aaaaaaaagaataaaaaaaaaatgatgaattgaaaaggtggtatggtgcactctcagtacaatctgctctgatgccgcatagttaagccagc
cccgacacccgccaacacccgctgacgcgccctgacgggcttgtctgctcccggcatccgcttacagacaagctgtgaccgtctccgggagctg
catgtgtcagaggttttcaccgtcatcaccgaaacgcgcgag-3’

pMAHS-GFP

5’-
acgaaagggcctcgtgatacgcctatttttataggttaatgtcatgataataatggtttcttagtatgatccaatatcaaaggaaatgatagcatt
gaaggatgagactaatccaattgaggagtggcagcatatagaacagctaaagggtagtgctgaaggaagcatacgataccccgcatggaat
gggataatatcacaggaggtactagactacctttcatcctacataaatagacgcatataagtacgcatttaagcataaacacgcactatgccgtt
cttctcatgtatatatatatacaggcaacacgcagatataggtgcgacgtgaacagtgagctgtatgtgcgcagctcgcgttgcattttcggaag
cgctcgttttcggaaacgctttgaagttcctattccgaagttcctattctctagaaagtataggaacttcagagcgcttttgaaaaccaaaagcgct
ctgaagacgcactttcaaaaaaccaaaaacgcaccggactgtaacgagctactaaaatattgcgaataccgcttccacaaacattgctcaaaa
gtatctctttgctatatatctctgtgctatatccctatataacctacccatccacctttcgctccttgaacttgcatctaaactcgacctctacatttttt
atgtttatctctagtattactctttagacaaaaaaattgtagtaagaactattcatagagtgaatcgaaaacaatacgaaaatgtaaacatttcct
atacgtagtatatagagacaaaatagaagaaaccgttcataatttctgaccaatgaagaatcatcaacgctatcactttctgttcacaaagtat
gcgcaatccacatcggtatagaatataatcggggatgcctttatcttgaaaaaatgcacccgcagcttcgctagtaatcagtaaacgcgggaag
tggagtcaggcttttttatggaagagaaaatagacacCaaagtagccttcttctaaccttaacggacctacagtgcaaaaagttatcaagaga
ctgcattatagagcgcacaaaggagaaaaaaagtaatctaagatgctttgttagaaaaatagcgctctcgggatgcatttttgtagaacaaaa
aagaagtatagattctttgttggtaaaatagcgctctcgcgttgcatttctgttctgtaaaaatgcagctcagattctttgtttgaaaaattagcgct
ctcgcgttgcatttttgttttacaaaaatgaagcacagattcttcgttggtaaaatagcgctttcgcgttgcatttctgttctgtaaaaatgcagctca
gattctttgtttgaaaaattagcgctctcgcgttgcatttttgttctacaaaatgaagcacagatgcttcgttcaggtggcacttttcggggaaatgt
gcgcggaacccctatttgtttatttttctaaatacattcaaatatgtatccgctcatgagacaataaccctgataaatgcttcaataatattgaaaa
aggaagagtatgagtattcaacatttccgtgtcgcccttattcccttttttgcggcattttgccttcctgtttttgctcacccagaaacgctggtgaaa
gtaaaagatgctgaagatcagttgggtgcacgagtgggttacatcgaactggatctcaacagcggtaagatccttgagagttttcgccccgaag
aacgttttccaatgatgagcacttttaaagttctgctatgtggcgcggtattatcccgtattgacgccgggcaagagcaactcggtcgccgcatac
actattctcagaatgacttggttgagtactcaccagtcacagaaaagcatcttacggatggcatgacagtaagagaattatgcagtgctgccata
accatgagtgataacactgcggccaacttacttctgacaacgatcggaggaccgaaggagctaaccgcttttttgcacaacatgggggatcatg
taactcgccttgatcgttgggaaccggagctgaatgaagccataccaaacgacgagcgtgacaccacgatgcctgtagcaatggcaacaacgt
tgcgcaaactattaactggcgaactacttactctagcttcccggcaacaattaatagactggatggaggcggataaagttgcaggaccacttct
gcgctcggcccttccggctggctggtttattgctgataaatctggagccggtgagcgtgggtctcgcggtatcattgcagcactggggccagatg
gtaagccctcccgtatcgtagttatctacacgacggggagtcaggcaactatggatgaacgaaatagacagatcgctgagataggtgcctcact
gattaagcattggtaactgtcagaccaagtttactcatatatactttagattgatttaaaacttcatttttaatttaaaaggatctaggtgaagatcc
tttttgataatctcatgaccaaaatcccttaacgtgagttttcgttccactgagcgtcagaccccgtagaaaagatcaaaggatcttcttgagatcc

tttttttctgcgcgtaatctgctgcttgcaaacaaaaaaaccaccgctaccagcggtggtttgtttgccggatcaagagctaccaactctttttccg
aaggtaactggcttcagcagagcgcagataccaaatactgttcttctagtgtagccgtagttaggccaccacttcaagaactctgtagcaccgcc
tacatacctcgctctgctaatcctgttaccagtggctgctgccagtggcgataagtcgtgtcttaccgggttggactcaagacgatagttaccgga
taaggcgcagcggtcgggctgaacggggggttcgtgcacacagcccagcttggagcgaacgacctacaccgaactgagatacctacagcgtg
agctatgagaaagcgccacgcttcccgaagggagaaaggcggacaggtatccggtaagcggcagggtcggaacaggagagcgcacgagg
gagcttccagggggaaacgcctggtatctttatagtcctgtcgggtttcgccacctctgacttgagcgtcgatttttgtgatgctcgtcaggggggc
ggagcctatggaaaaacgccagcaacgcggcctttttacggttcctggccttttgctggccttttgctcacatgttctttcctgcgttatcccctgatt
ctgtggataaccgtattaccgcctttgagtgagctgataccgctcgccgcagccgaacgaccgagcgcagcgagtcagtgagcgaggaagcgg
aagagcgcccaatacgcaaaccgcctctccccgcgcgttggccgattcattaatgcagctggcacgacaggtttcccgactggaaagcgggca
gtgagcgcaacgcaattaatgtgagttagctcactcattaggcaccccaggctttacactttatgcttccggctcgtatgttgtgtggaattgtgag
cggataacaatttcacacaggaaacagctatgaccatgattacgccaagcgcgcaattaaccctcactaaagggaacaaaagctggAGCTC
Agccacaatagaagctttctaactgatctatccaaaactgaaaattacattcttgattaggtttatcacaggcaaatgtaatttgtggtattttgcc
gttcaaaatctgtagaatttctcattggtcacattacaacctgaaaatactttatctacaatcataccattcttataacatgtccccttaatactag
gatcaggcatgaacgcatcacagacaaaatcttcttgacaaacgtcacaattgatccctccccatccgttatcacaatgacaggtgtcattttgtg
ctcttatgggacgatccttattaccgctttcatccggtgatagaccgccacagaggggcagagagcaatcatcacctgcaaacccttctatacac
tcacatctaccagtgtacgaattgcattcagaaaactgtttgcattcaaaaataggtagcatacaattaaaacatggcgggcatgtatcattgcc
cttatcttgtgcagttagacgcgaatttttcgaagaagtaccttcaaagaatggggtcttatcttgttttgcaagtaccactgagcaggataataat
agaaatgataatatactatagtagagataacgtcgatgacttcccatactgtaattgcttttagttgtgtatttttagtgtgcaagtttctgtaaatc
gattaatttttttttctttcctctttttattaaccttaatttttattttagattcctgacttcaactcaagacgcacagatattataacatctgcataatag
gcatttgcaagaattactcgtgagtaaggaaagagtgaggaactatcgcatacctgcatttaaagatgccgatttgggcgcgaatcctttattttg
gcttcaccctcatactattatcagggccagaaaaaggaagtgtttccctccttcttgaattgatgttaccctcataaagcacgtggcctcttatcga
gaaagaaattaccgtcgctcgtgatttgtttgcaaaaagaacaaaactgaaaaaacccagacacgctCgacttcctgtcttcctattgattgcag
cttccaatttcgtcacacaacaaggtcctagcgacggctcacaggttttgtaacaagcaatcgaaggttctggaatggcgggaaagggtttagt
accacatgctatgatgcccactgtgatctccagagcaaagttcgttcgatcgtactgttactctctctctttcaaacagaattgtccgaatcgtgtg
acaacaacagcctgttctcacacactcttttcttctaaccaagggggtggtttagtttagtagaacctcgtgaaacttacatttacatatatataaa
cttgcataaattggtcaatgcaagaaatacatatttggtcttttctaattcgtagtttttcaagttcttagatgctttctttttctctttttacagatcaT
CAAGGAAGTAATTATCTACTTTTTACAACAAATATAAAACAatgtccagatacctgctgcgcgatgtccaggctgtattacgcg
gagttcgcaaagtggccgagagtagcttaaagctggagacggagaaagtcagtctgcggcttggtgactttcggtcacagccttcccttcgcag
tgtgcctgcttccctcacaagtcgatcacaggcatttagcctacaggagatagctgctcgtgccggagttgttctgcgaggagtgcaacaacagt
tccgaaacgtcactggagtgaatgccgctcctgttgtagcctttgataatggatcagttctatacagtgaaagaatccactcgcagagttcgcag
aagcaggccccgactacagtaccaacaggatccgtcagcaattcccctcaaccggaaggaaaggcaaacgaagctgctgaacgcgcaaaac
agtttatgaatcctccagttgcgccaatggatcctgtcgacaagaatgaatttgtcgccatgccggagatgggtcgtagtaatggaaatggaga
aaacaaacaagctgctgatttcatgaaaaaccaaggtgacaccgatatggattcccagtacgcgcctgattcatcgaagaacacgaaatcggt
tcccacgaaggaaatcgttgctgaagatggttcgatgagcattgaggatatcaagaaagctacgcaggttactcctggagttgcagttaaaaac
gagggtgttgatccaccggtcatggtgagcaagggcgccgagctgttcaccggcatcgtgcccatcctgatcgagctgaatggcgatgtgaatg
gccacaagttcagcgtgagcggcgagggcgagggcgatgccacctacggcaagctgaccctgaagttcatctgcaccaccggcaagctgcct
gtgccctggcccaccctggtgaccaccctgagctacggcgtgcagtgcttctcacgctaccccgatcacatgaagcagcacgacttcttcaaga
gcgccatgcctgagggctacatccaggagcgcaccatcttcttcgaggatgacggcaactacaagtcgcgcgccgaggtgaagttcgagggcg
ataccctggtgaatcgcatcgagctgaccggcaccgatttcaaggaggatggcaacatcctgggcaataagatggagtacaactacaacgcc
cacaatgtgtacatcatgaccgacaaggccaagaatggcatcaaggtgaacttcaagatccgccacaacatcgaggatggcagcgtgcagct
ggccgaccactaccagcagaatacccccatcggcgatggccctgtgctgctgcccgataaccactacctgtccacccagagcgccctgtccaa
ggaccccaacgagaagcgcgatcacatgatctacttcggcttcgtgaccgccgccgccatcacccacggcatggatgagctgtacaagtaaAT
ATTGAATTGAATTGAAATCGATAGATCAATTTTTTTCTTTTCTCTTTCCCCATCCTTTACGCTAAAATAATAGTTTA
TTTTATTTTTTGAATATTTTTTATTTATATACGTATATATAGACTATTATTTATCTTTTAATGATTATTAAGATTTTTATT
AAAAAAAAATTCGCTCCTCTTTTAATGCCTTTATGCAGTTTTTTTTTCCCATTCGATATTTCTATGTTCGGGTTCA
GCGTATTTTAAGTTTAATAACTCGAAAATTCTGCGTTCGTTAAAGCTTTCGAGAAGGATATTATTTCGAAATAAA
CCGTGTTGTGTAAGCTTGAAGCCTTTTTGCGCTGCCAATATTCTTATCCATCTATTGTACTCTTTAGATCCAGTAT
AGTGTATTCTTCCTGCTCCAAGCTCATCCCACTTGCAACAAAAAAAGTCTAATCTTCTGCAATAATTTCCATCCTT

GGCATTCAGAGACATATATTGGTCAATCGGTTTTAATTTGgtacccaattcgccctatagtgagtcgtattacgcgcgctcactg
gccgtcgttttacaacgtcgtgactgggaaaaccctggcgttacccaacttaatcgccttgcagcacatccccctttcgccagctggcgtaatagc
gaagaggcccgcaccgatcgcccttcccaacagttgcgcagcctgaatggcgaatggacgcgccctgtagcggcgcattaagcgcggcgggt
gtggtggttacgcgcagcgtgaccgctacacttgccagcgccctagcgcccgctcctttcgctttcttcccttcctttctcgccacgttcgccggcttt
ccccgtcaagctctaaatcgggggctccctttagggttccgatttagtgctttacggcacctcgaccccaaaaaacttgattagggtgatggttca
cgtagtgggccatcgccctgatagacggtttttcgccctttgacgttggagtccacgttctttaatagtggactcttgttccaaactggaacaacac
tcaaccctatctcggtctattcttttgatttataagggattttgccgatttcggcctattggttaaaaaatgagctgatttaacaaaaatttaacgcg
aattttaacaaaatattaacgcttacaatttcctgatgcggtattttctccttacgcatctgtgcggtatttcacaccgcatagggtaataactgata
taattaaattgaagctctaatttgtgagtttagtatacatgcatttacttataatacagttttttagttttgctggccgcatcttctcaaatatgcttccc
agcctgcttttctgtaacgttcaccctctaccttagcatcccttccctttgcaaatagtcctcttccaacaataataatgtcagatcctgtagagacc
acatcatccacggttctatactgttgacccaatgcgtctcccttgtcatctaaacccacaccgggtgtcataatcaaccaatcgtaaccttcatctc
ttccacccatgtctctttgagcaataaagccgataacaaaatctttgtcgctcttcgcaatgtcaacagtacccttagtatattctccagtagatag
ggagcccttgcatgacaattctgctaacatcaaaaggcctctaggttcctttgttacttcttctgccgcctgcttcaaaccgctaacaatacctggg
cccaccacaccgtgtgcattcgtaatgtctgcccattctgctattctgtatacacccgcagagtactgcaatttgactgtattaccaatgtcagcaa
attttctgtcttcgaagagtaaaaaattgtacttggcggataatgcctttagcggcttaactgtgccctccatggaaaaatcagtcaagatatcca
catgtgtttttagtaaacaaattttgggacctaatgcttcaactaactccagtaattccttggtggtacgaacatccaatgaagcacacaagtttgt
ttgcttttcgtgcatgatattaaatagcttggcagcaacaggactaggatgagtagcagcacgttccttatatgtagctttcgacatgatttatcttc
gtttcctgcaggtttttgttctgtgcagttgggttaagaatactgggcaatttcatgtttcttcaacactacatatgcgtatatataccaatctaagtc
tgtgctccttccttcgttcttccttctgttcggagattaccgaatcaaaaaaatttcaaggaaaccgaaatcaaaaaaaagaataaaaaaaaaat
gatgaattgaaaaggtggtatggtgcactctcagtacaatctgctctgatgccgcatagttaagccagccccgacacccgccaacacccgctga
cgcgccctgacgggcttgtctgctcccggcatccgcttacagacaagctgtgaccgtctccgggagctgcatgtgtcagaggttttcaccgtcatc
accgaaacgcgcgag-3’

pRvLEAM-GFP

5’-
acgaaagggcctcgtgatacgcctatttttataggttaatgtcatgataataatggtttcttagtatgatccaatatcaaaggaaatgatagcatt
gaaggatgagactaatccaattgaggagtggcagcatatagaacagctaaagggtagtgctgaaggaagcatacgataccccgcatggaat
gggataatatcacaggaggtactagactacctttcatcctacataaatagacgcatataagtacgcatttaagcataaacacgcactatgccgtt
cttctcatgtatatatatatacaggcaacacgcagatataggtgcgacgtgaacagtgagctgtatgtgcgcagctcgcgttgcattttcggaag
cgctcgttttcggaaacgctttgaagttcctattccgaagttcctattctctagaaagtataggaacttcagagcgcttttgaaaaccaaaagcgct
ctgaagacgcactttcaaaaaaccaaaaacgcaccggactgtaacgagctactaaaatattgcgaataccgcttccacaaacattgctcaaaa
gtatctctttgctatatatctctgtgctatatccctatataacctacccatccacctttcgctccttgaacttgcatctaaactcgacctctacattttttt
atgtttatctctagtattactctttagacaaaaaaattgtagtaagaactattcatagagtgaatcgaaaacaatacgaaaatgtaaacatttcct
atacgtagtatatagagacaaaatagaagaaaccgttcataattttctgaccaatgaagaatcatcaacgctatcactttctgttcacaaagtat
gcgcaatccacatcggtatagaatataatcggggatgcctttatcttgaaaaaatgcacccgcagcttcgctagtaatcagtaaacgcgggaag
tggagtcaggctttttttatggaagagaaaatagacaccaaagtagccttcttctaaccttaacggacctacagtgcaaaaagttatcaagaga
ctgcattatagagcgcacaaaggagaaaaaaagtaatctaagatgctttgttagaaaaatagcgctctcgggatgcatttttgtagaacaaaa
aagaagtatagattctttgttggtaaaatagcgctctcgcgttgcatttctgttctgtaaaaatgcagctcagattctttgtttgaaaaattagcgct
ctcgcgttgcatttttgttttacaaaaatgaagcacagattcttcgttggtaaaatagcgctttcgcgttgcatttctgttctgtaaaaatgcagctca
gattctttgtttgaaaaattagcgctctcgcgttgcatttttgttctacaaaatgaagcacagatgcttcgttcaggtggcacttttcggggaaatgt
gcgcggaacccctatttgtttatttttctaaatacattcaaatatgtatccgctcatgagacaataaccctgataaatgcttcaataatattgaaaa
aggaagagtatgagtattcaacatttccgtgtcgcccttattcccttttttgcggcattttgccttcctgtttttgctcacccagaaacgctggtgaaa
gtaaaagatgctgaagatcagttgggtgcacgagtgggttacatcgaactggatctcaacagcggtaagatccttgagagttttcgccccgaag
aacgttttccaatgatgagcacttttaaagttctgctatgtggcgcggtattatcccgtattgacgccgggcaagagcaactcggtcgccgcatac
actattctcagaatgacttggttgagtactcaccagtcacagaaaagcatcttacggatggcatgacagtaagagaattatgcagtgctgccata
accatgagtgataacactgcggccaacttacttctgacaacgatcggaggaccgaaggagctaaccgcttttttgcacaacatgggggatcatg

taactcgccttgatcgttgggaaccggagctgaatgaagccataccaaacgacgagcgtgacaccacgatgcctgtagcaatggcaacaacgt
tgcgcaaactattaactggcgaactacttactctagcttcccggcaacaattaatagactggatggaggcggataaagttgcaggaccacttct
gcgctcggcccttccggctggctggtttattgctgataaatctggagccggtgagcgtgggtctcgcggtatcattgcagcactggggccagatg
gtaagccctcccgtatcgtagttatctacacgacggggagtcaggcaactatggatgaacgaaatagacagatcgctgagataggtgcctcact
gattaagcattggtaactgtcagaccaagtttactcatatatactttagattgatttaaaacttcatttttaatttaaaaggatctaggtgaagatcc
tttttgataatctcatgaccaaaatcccttaacgtgagttttcgttccactgagcgtcagaccccgtagaaaagatcaaaggatcttcttgagatcc
tttttttctgcgcgtaatctgctgcttgcaaacaaaaaaaccaccgctaccagcggtggtttgtttgccggatcaagagctaccaactctttttccg
aaggtaactggcttcagcagagcgcagataccaaatactgttcttctagtgtagccgtagttaggccaccacttcaagaactctgtagcaccgcc
tacatacctcgctctgctaatcctgttaccagtggctgctgccagtggcgataagtcgtgtcttaccgggttggactcaagacgatagttaccgga
taaggcgcagcggtcgggctgaacggggggttcgtgcacacagcccagcttggagcgaacgacctacaccgaactgagatacctacagcgtg
agctatgagaaagcgccacgcttcccgaagggagaaaggcggacaggtatccggtaagcggcagggtcggaacaggagagcgcacgagg
gagcttccagggggaaacgcctggtatctttatagtcctgtcgggtttcgccacctctgacttgagcgtcgatttttgtgatgctcgtcaggggggc
ggagcctatggaaaaacgccagcaacgcggcctttttacggttcctggccttttgctggccttttgctcacatgttctttcctgcgttatcccctgatt
ctgtggataaccgtattaccgcctttgagtgagctgataccgctcgccgcagccgaacgaccgagcgcagcgagtcagtgagcgaggaagcgg
aagagcgcccaatacgcaaaccgcctctccccgcgcgttggccgattcattaatgcagctggcacgacaggtttcccgactggaaagcgggca
gtgagcgcaacgcaattaatgtgagttagctcactcattaggcaccccaggctttacactttatgcttccggctcgtatgttgtgtggaattgtgag
cggataacaatttcacacaggaaacagctatgaccatgattacgccaagcgcgcaattaaccctcactaaagggaacaaaagctggAGCTC
Agccacaatagaagctttctaactgatctatccaaaactgaaaattacattcttgattaggtttatcacaggcaaatgtaatttgtggtattttgcc
gttcaaaatctgtagaattttctcattggtcacattacaacctgaaaatactttatctacaatcataccattcttataacatgtccccttaatactag
gatcaggcatgaacgcatcacagacaaaatcttcttgacaaacgtcacaattgatccctccccatccgttatcacaatgacaggtgtcattttgtg
ctcttatgggacgatccttattaccgctttcatccggtgatagaccgccacagaggggcagagagcaatcatcacctgcaaacccttctatacac
tcacatctaccagtgtacgaattgcattcagaaaactgtttgcattcaaaaataggtagcatacaattaaaacatggcgggcatgtatcattgcc
cttatcttgtgcagttagacgcgaatttttcgaagaagtaccttcaaagaatggggtcttatcttgttttgcaagtaccactgagcaggataataat
agaaatgataatatactatagtagagataacgtcgatgacttcccatactgtaattgcttttagttgtgtatttttagtgtgcaagtttctgtaaatc
gattaattttttttctttcctcttttattaaccttaatttttattttagattcctgacttcaactcaagacgcacagatattataacatctgcataatag
gcatttgcaagaattactcgtgagtaaggaaagagtgaggaactatcgcatacctgcatttaaagatgccgatttgggcgcgaatcctttattttg
gcttcaccctcatactattatcagggccagaaaaaggaagtgtttccctccttcttgaattgatgttaccctcataaagcacgtggcctcttatcga
gaaagaaattaccgtcgctcgtgatttgtttgcaaaaagaacaaaactgaaaaaacccagacacgctcgacttcctgtcttcctattgattgcag
cttccaatttcgtcacacaacaaggtcctagcgacggctcacaggttttgtaacaagcaatcgaaggttctggaatggcgggaaagggtttagt
accacatgctatgatgcccactgtgatctccagagcaaagttcgttcgatcgtactgttactctctctctttcaaacagaattgtccgaatcgtgtg
acaacaacagcctgttctcacacactcttttcttctaaccaagggggtggtttagtttagtagaacctcgtgaaacttacatttacatatatataaa
cttgcataaattggtcaatgcaagaaatacatatttggtcttttctaattcgtagtttttcaagttcttagatgctttctttttctcttttttacagatcaT
CAAGGAAGTAATTATCTACTTTTTACAACAAATATAAAACAatgtttctcgcccgaaacgctggacgcgctggatatcgaggtg
ttgtagcttaccagcaagcagcctctttctctgtttcatcagctaaggctgctggatcacggtcaagcggtggctccgacgctggcgattatgcta
gggaggccgcggaacatgccaaagctggattgaaggatctcaagaatgaagcctcgtggaaagccaaaggtgtcgctaaccaagccgctgg
agcattcgaacgagcgaaggatacagtgaaggaaggtgtccatgacatgaagcgcagtggaagccgcgtcttcgaacaaggacaggaggaa
gtagaggctggtgctcaacacgcaaaggctggctatcagagtgccaagaatgctgcgcaagacactgccgcaactcttaaagacaaagccgg
cagcgcctggaatcaagcgaaacacgttgtggaagataagggagaggatgttgttgaagcagtcaaggacactgcttcaaaagtctggggca
aagccaaacatgtggccgaggacgttaaggagaacgcgcaatcaccaggtggcattgcggataaggcctccgatgtgtggtcagcggccaag
gataaagctgctgatgtcttgtcgggagctaagcacactgctgagaacctagctcacaaagcccaagccgccatccacgatgctacagcttctt
caggctcgcagtcgcagtcgcagtcccagtctcagtatcgacaaggacaacaacagggaagacaagatcagcagcaaagcaagagccagtg
gggacagactagcccgcagtctccggatggattccgccctcaagccggccagggaccacaaggaggcaagggacctggtcaagctggcggt
cgacgagatccaccggtcatggtgagcaagggcgccgagctgttcaccggcatcgtgcccatcctgatcgagctgaatggcgatgtgaatggc
cacaagttcagcgtgagcggcgagggcgagggcgatgccacctacggcaagctgaccctgaagttcatctgcaccaccggcaagctgcctgt
gccctggcccaccctggtgaccaccctgagctacggcgtgcagtgcttctcacgctaccccgatcacatgaagcagcacgacttcttcaagagc
gccatgcctgagggctacatccaggagcgcaccatcttcttcgaggatgacggcaactacaagtcgcgcgccgaggtgaagttcgagggcgat
accctggtgaatcgcatcgagctgaccggcaccgatttcaaggaggatggcaacatcctgggcaataagatggagtacaactacaacgccca
caatgtgtacatcatgaccgacaaggccaagaatggcatcaaggtgaacttcaagatccgccacaacatcgaggatggcagcgtgcagctgg

ccgaccactaccagcagaatacccccatcggcgatggccctgtgctgctgcccgataaccactacctgtccacccagagcgccctgtccaagg
accccaacgagaagcgcgatcacatgatctacttcggcttcgtgaccgccgccgccatcacccacggcatggatgagctgtacaagtaaATAT
TGAATTGAATTGAAATCGATAGATCAATTTTTTTCTTTTCTCTTTCCCCATCCTTTACGCTAAAATAATAGTTTATT
TTATTTTTTGAATATTTTTTATTTATATACGTATATATAGACTATTATTTATCTTTTAATGATTATTAAGATTTTTATTA
AAAAAAAATTCGCTCCTCTTTTAATGCCTTTATGCAGTTTTTTTTTCCCATTCGATATTTCTATGTTCGGGTTCAG
CGTATTTTAAGTTTAATAACTCGAAAATTCTGCGTTCGTTAAAGCTTTCGAGAAGGATATTATTTCGAAATAAAC
CGTGTTGTGTAAGCTTGAAGCCTTTTTGCGCTGCCAATATTCTTATCCATCTATTGTACTCTTTAGATCCAGTATA
GTGTATTCTTCCTGCTCCAAGCTCATCCCACTTGCAACAAAAAAAGTCTAATCTTCTGCAATAATTTCCATCCTTG
GCATTCAGAGACATATATTGGTCAATCGGTTTTAATTTGgtacccaattcgccctatagtgagtcgtattacgcgcgctcactggc
cgtcgttttacaacgtcgtgactgggaaaaccctggcgttacccaacttaatcgccttgcagcacatccccctttcgccagctggcgtaatagcga
agaggcccgcaccgatcgcccttcccaacagttgcgcagcctgaatggcgaatggacgcgccctgtagcggcgcattaagcgcggcgggtgtg
gtggttacgcgcagcgtgaccgctacacttgccagcgccctagcgcccgctcctttcgctttcttcccttcctttctcgccacgttcgccggctttccc
cgtcaagctctaaatcgggggctccctttagggttccgatttagtgctttacggcacctcgaccccaaaaaacttgattagggtgatggttcacgt
agtgggccatcgccctgatagacggtttttcgccctttgacgttggagtccacgttctttaatagtggactcttgttccaaactggaacaacactca
accctatctcggtctattcttttgatttataagggattttgccgatttcggcctattggttaaaaaatgagctgatttaacaaaaatttaacgcgaatt
ttaacaaaatattaacgcttacaatttcctgatgcggtattttctccttacgcatctgtgcggtatttcacaccgcatagggtaataactgatataat
taaattgaagctctaatttgtgagtttagtatacatgcatttacttataatacagtttttagttttgctggccgcatcttctcaaatatgcttcccagc
ctgcttttctgtaacgttcaccctctaccttagcatcccttccctttgcaaatagtcctcttccaacaataataatgtcagatcctgtagagaccaca
tcatccacggttctatactgttgacccaatgcgtctcccttgtcatctaaacccacaccgggtgtcataatcaaccaatcgtaaccttcatctcttcc
acccatgtctctttgagcaataaagccgataacaaaatctttgtcgctcttcgcaatgtcaacagtacccttagtatattctccagtagatagggа
gcccttgcatgacaattctgctaacatcaaaaggcctctaggttcctttgttacttcttctgccgcctgcttcaaaccgctaacaatacctgggccc
accacaccgtgtgcattcgtaatgtctgcccattctgctattctgtatacacccgcagagtactgcaatttgactgtattaccaatgtcagcaaattt
tctgtcttcgaagagtaaaaaattgtacttggcggataatgcctttagcggcttaactgtgccctccatggaaaaatcagtcaagatatccacat
gtgtttttagtaaacaaattttgggacctaatgcttcaactaactccagtaattccttggtggtacgaacatccaatgaagcacacaagtttgtttg
cttttcgtgcatgatattaaatagcttggcagcaacaggactaggatgagtagcagcacgttccttatatgtagctttcgacatgatttatcttcgtt
tcctgcaggtttttgttctgtgcagttgggttaagaatactgggcaatttcatgtttcttcaacactacatatgcgtatatataccaatctaagtctgt
gctccttccttcgttcttccttctgttcggagattaccgaatcaaaaaaatttcaaggaaaccgaaatcaaaaaaaagaataaaaaaaaaatga
tgaattgaaaaggtggtatggtgcactctcagtacaatctgctctgatgccgcatagttaagccagccccgacacccgccaacacccgctgacg
cgccctgacgggcttgtctgctcccggcatccgcttacagacaagctgtgaccgtctccgggagctgcatgtgtcataggttttcaccgtcatcac
cgaaacgcgcgag-3’

pIW14

5’-
acgaaagggcctcgtgatacgcctatttttataggttaatgtcatgataataatggtttcttagtatgatccaatatcaaaggaaatgatagcatt
gaaggatgagactaatccaattgaggagtggcagcatatagaacagctaaagggtagtgctgaaggaagcatacgataccccgcatggaat
gggataatatcacaggaggtactagactacctttcatcctacataaatagacgcatataagtacgcatttaagcataaacacgcactatgccgtt
cttctcatgtatatatatatacaggcaacacgcagatataggtgcgacgtgaacagtgagctgtatgtgcgcagctcgcgttgcattttcggaag
cgctcgttttcggaaacgctttgaagttcctattccgaagttcctattctctagaaagtataggaacttcagagcgcttttgaaaaccaaaagcgct
ctgaagacgcactttcaaaaaaccaaaaacgcaccggactgtaacgagctactaaaatattgcgaataccgcttccacaaacattgctcaaaa
gtatctctttgctatatatctctgtgctatatccctatataacctacccatccacctttcgctccttgaacttgcatctaaactcgacctctacattttt
atgtttatctctagtattactctttagacaaaaaaattgtagtaagaactattcatagagtgaatcgaaaacaatacgaaaatgtaaacatttcct
atacgtagtatatagagacaaaatagaagaaaccgttcataattttctgaccaatgaagaatcatcaacgctatcactttctgttcacaaagtat
gcgcaatccacatcggtatagaatataatcggggatgcctttatcttgaaaaaatgcacccgcagcttcgctagtaatcagtaaacgcgggaag
tggagtcaggctttttttatggaagagaaaatagacaccaaagtagccttcttctaaccttaacggacctacagtgcaaaaagttatcaagaga
ctgcattatagagcgcacaaaggagaaaaaaagtaatctaagatgctttgttagaaaaatagcgctctcgggatgcattttgtagaacaaaa
aagaagtatagattctttgttggtaaaatagcgctctcgcgttgcatttctgttctgtaaaaatgcagctcagattctttgtttgaaaaattagcgct

ctcgcgttgcatttttgttttacaaaaatgaagcacagattcttcgttggtaaaatagcgctttcgcgttgcatttctgttctgtaaaaatgcagctca
gattctttgtttgaaaaattagcgctctcgcgttgcatttttgttctacaaaatgaagcacagatgcttcgttcaggtggcacttttcggggaaatgt
gcgcggaacccctatttgtttatttttctaaatacattcaaatatgtatccgctcatgagacaataaccctgataaatgcttcaataatattgaaaa
aggaagagtatgagtattcaacatttccgtgtcgcccttattcccttttttgcggcattttgccttcctgtttttgctcacccagaaacgctggtgaaa
gtaaaagatgctgaagatcagttgggtgcacgagtgggttacatcgaactggatctcaacagcggtaagatccttgagagttttcgccccgaag
aacgttttccaatgatgagcacttttaaagttctgctatgtggcgcggtattatcccgtattgacgccgggcaagagcaactcggtcgccgcatac
actattctcagaatgacttggttgagtactcaccagtcacagaaaagcatcttacggatggcatgacagtaagagaattatgcagtgctgccata
accatgagtgataacactgcggccaacttacttctgacaacgatcggaggaccgaaggagctaaccgcttttttgcacaacatgggggatcatg
taactcgccttgatcgttgggaaccggagctgaatgaagccataccaaacgacgagcgtgacaccacgatgcctgtagcaatggcaacaacgt
tgcgcaaactattaactggcgaactacttactctagcttcccggcaacaattaatagactggatggaggcggataaagttgcaggaccacttct
gcgctcggcccttccggctggctggtttattgctgataaatctggagccggtgagcgtgggtctcgcggtatcattgcagcactggggccagatg
gtaagccctcccgtatcgtagttatctacacgacggggagtcaggcaactatggatgaacgaaatagacagatcgctgagataggtgcctcact
gattaagcattggtaactgtcagaccaagtttactcatatatactttagattgatttaaaacttcatttttaatttaaaaggatctaggtgaagatcc
tttttgataatctcatgaccaaaatcccttaacgtgagttttcgttccactgagcgtcagaccccgtagaaaagatcaaaggatcttcttgagatcc
tttttttctgcgcgtaatctgctgcttgcaaacaaaaaaaccaccgctaccagcggtggtttgtttgccggatcaagagctaccaactctttttccg
aaggtaactggcttcagcagagcgcagataccaaatactgtccttctagtgtagccgtagttaggccaccacttcaagaactctgtagcaccgc
ctacatacctcgctctgctaatcctgttaccagtggctgctgccagtggcgataagtcgtgtcttaccgggttggactcaagacgatagttaccgg
ataaggcgcagcggtcgggctgaacggggggttcgtgcacacagcccagcttggagcgaacgacctacaccgaactgagatacctacagcgt
gagctatgagaaagcgccacgcttcccgaagggagaaaggcggacaggtatccggtaagcggcagggtcggaacaggagagcgcacgag
ggagcttccagggggaaacgcctggtatctttatagtcctgtcgggtttcgccacctctgacttgagcgtcgatttttgtgatgctcgtcaggggg
gcggagcctatggaaaaacgccagcaacgcggcctttttacggttcctggccttttgctggccttttgctcacatgttctttcctgcgttatcccctg
attctgtggataaccgtattaccgcctttgagtgagctgataccgctcgccgcagccgaacgaccgagcgcagcgagtcagtgagcgaggaag
cggaagagcgcccaatacgcaaaccgcctctccccgcgcgttggccgattcattaatgcagctggcacgacaggtttcccgactggaaagcgg
gcagtgagcgcaacgcaattaatgtgagttacctcactcattaggcaccccaggctttacactttatgcttccggctcctatgttgtgtggaattgt
gagcggataacaatttcacacaggaaacagctatgaccatgattacgccaagcgcgcaattaaccctcactaaagggaacaaaagctggAG
CTCAgccacaatagaagctttctaactgatctatccaaaactgaaaattacattcttgattaggtttatcacaggcaaatgtaatttgtggtatttt
gccgttcaaaatctgtagaattttctcattggtcacattacaacctgaaaatactttatctacaatcataccattcttataacatgtccccttaatact
aggatcaggcatgaacgcatcacagacaaaatcttcttgacaaacgtcacaattgatccctccccatccgttatcacaatgacaggtgtcatttt
gtgctcttatgggacgatccttattaccgctttcatccggtgatagaccgccacagaggggcagagagcaatcatcacctgcaaacccttctata
cactcacatctaccagtgtacgaattgcattcagaaaactgtttgcattcaaaaataggtagcatacaattaaaacatggcgggcatgtatcatt
gcccttatcttgtgcagttagacgcgaattttcgaagaagtaccttcaaagaatggggtcttatcttgttttgcaagtaccactgagcaggataat
aatagaaatgataatatactatagtagagataacgtcgatgacttcccatactgtaattgcttttagttgtgtatttttagtgtgcaagtttctgtaa
atcgattaatttttttttctttcctcttttattaaccttaatttttattttagattcctgacttcaactcaagacgcacagatattataacatctgcataat
aggcatttgcaagaattactcgtgagtaaggaaagagtgaggaactatcgcatacctgcatttaaagatgccgatttgggcgcgaatcctttatt
ttggcttcaccctcatactattatcagggccagaaaaaggaagtgtttccctccttcttgaattgatgttaccctcataaagcacgtggcctcttatc
gagaaagaaattaccgtcgctcgtgatttgtttgcaaaaagaacaaaactgaaaaaacccagacacgctcgacttcctgtcttcctattgattgc
agcttccaatttcgtcacacaacaaggtcctagcgacggctcacaggttttgtaacaagcaatcgaaggttctggaatggcgggaaagggttta
gtaccacatgctatgatgcccactgtgatctccagagcaaagttcgttcgatcgtactgttactctctctctttcaaacagaattgtccgaatcgtgt
gacaacaacagcctgttctcacacactcttttcttctaaccaagggggtggtttagtttagtagaacctcgtgaaacttacatttacatatatataa
acttgcataaattggtcaatgcaagaaatacatatttggtcttttctaattcgtagtttttcaagttcttagatgctttctttttctctttttacagatca
CCgcggtggcggccgctctagaactagtATTGAATTGAATTGAAATCGATAGATCAATTTTTTTCTTTTCTCTTTCCCCAT
CCTTTACGCTAAAATAATAGTTTATTTTATTTTTTGAATATTTTTTATTTATATACGTATATATAGACTATTATTTATCT
TTTAATGATTATTAAGATTTTTATTAAAAAAAAATTCGCTCCTCTTTTAATGCCTTTATGCAGTTTTTTTTTCCCAT
TCGATATTTCTATGTTCGGGTTCAGCGTATTTTAAGTTTAATAACTCGAAAATTCTGCGTTCGTTAAAGCTTTCG
AGAAGGATATTATTTCGAAATAAACCGTGTTGTGTAAGCTTGAAGCCTTTTTGCGCTGCCAATATTCTTATCCAT
CTATTGTACTCTTTAGATCCAGTATAGTGTATTCTTCCTGCTCCAAGCTCATCCCACTTGCAACAAAAAAAGTCTA
ATCTTCTGCAATAATTTCCATCCTTGGCATTCAGAGACATATATTGGTCAATCGGTTTTAATTTggtacccaattcgccc
tatagtgagtcgtattacgcgcgctcactggccgtcgttttacaacgtcgtgactgggaaaaccctggcgttacccaacttaatcgccttgcagca

catccccctttcgccagctggcgtaatagcgaagaggcccgcaccgatcgcccttcccaacagttgcgcagcctgaatggcgaatggcgcgac
gcgccctgtagcggcgcattaagcgcggcgggtgtggtggttacgcgcagcgtgaccgctacacttgccagcgccctagcgcccgctcctttcg
ctttcttcccttcctttctcgccacgttcgccggctttccccgtcaagctctaaatcgggggctccctttagggttccgatttagtgctttacggcacct
cgaccccaaaaaacttgattagggtgatggttcacgtagtgggccatcgccctgatagacggtttttcgccctttgacgttggagtccacgttcttt
aatagtggactcttgttccaaactggaacaacactcaaccctatctcggtctattcttttgatttataagggattttgccgatttcggcctattggtta
aaaaatgagctgatttaacaaaaatttaacgcgaattttaacaaaatattaacgtttacaatttcctgatgcggtattttctccttacgcatctgtgc
ggtatttcacaccgcatagggtaataactgatataattaaattgaagctctaatttgtgagtttagtatacatgcatttacttataatacagtttttta
gttttgctggccgcatcttctcaaatatgcttcccagcctgcttttctgtaacgttcaccctctaccttagcatcccttccctttgcaaatagtcctcttc
caacaataataatgtcagatcctgtagagaccacatcatccacggttctatactgttgacccaatgcgtctcccttgtcatctaaacccacaccgg
gtgtcataatcaaccaatcgtaaccttcatctcttccacccatgtctctttgagcaataaagccgataacaaaatctttgtcgctcttcgcaatgtc
aacagtacccttagtatattctccagtagatagggagcccttgcatgacaattctgctaacatcaaaaggcctctaggttcctttgttacttcttctg
ccgcctgcttcaaaccgctaacaatacctgggcccaccacaccgtgtgcattcgtaatgtctgcccattctgctattctgtatacacccgcagagt
actgcaatttgactgtattaccaatgtcagcaaattttctgtcttcgaagagtaaaaaattgtacttggcggataatgcctttagcggcttaactgt
gccctccatggaaaaatcagtcaagatatccacatgtgtttttagtaaacaaattttgggacctaatgcttcaactaactccagtaattccttggtg
gtacgaacatccaatgaagcacacaagtttgtttgcttttcgtgcatgatattaaatagcttggcagcaacaggactaggatgagtagcagcacg
ttccttatatgtagctttcgacatgatttatcttcgtttcctgcaggtttttgttctgtgcagttgggttaagaatactgggcaatttcatgtttcttcaac
actacatatgcgtatatataccaatctaagtctgtgctccttccttcgttcttccttctgttcggagattaccgaatcaaaaaaatttcaaagaaacc
gaaatcaaaaaaaagaataaaaaaaaaaatgatgaattgaattgaaaagctgtggtatggtgcactctcagtacaatctgctctgatgccgcat
agttaagccagccccgacacccgccaacacccgctgacgcgccctgacgggcttgtctgctcccggcatccgcttacagacaagctgtgaccgt
ctccgggagctgcatgtgtcagaggttttcaccgtcatcaccgaaacgcgcgag-3’
